# The Benefit of Slice Timing Correction in Common fMRI Preprocessing Pipelines

**DOI:** 10.3389/fnins.2019.00821

**Published:** 2019-08-20

**Authors:** David B. Parker, Qolamreza R. Razlighi

**Affiliations:** ^1^Department of Biomedical Engineering, Columbia University, New York, NY, United States; ^2^Department of Neurology, College of Physicians and Surgeons, Columbia University, New York, NY, United States; ^3^Taub Institute for Research on Alzheimer’s Disease and the Aging Brain, Columbia University, New York, NY, United States

**Keywords:** slice timing correction, fMRI — functional magnetic resonance imaging, motion correction, preprocessing algorithms, interleaved 2D multislice sequence

## Abstract

Due to the nature of fMRI acquisition protocols, slices cannot be acquired simultaneously, and as a result, are temporally misaligned from each other. To correct from this misalignment, preprocessing pipelines often incorporate slice timing correction (STC). However, evaluating the benefits of STC is challenging because it (1) is dependent on slice acquisition parameters, (2) interacts with head movement in a non-linear fashion, and (3) significantly changes with other preprocessing steps, fMRI experimental design, and fMRI acquisition parameters. Presently, the interaction of STC with various scan conditions has not been extensively examined. Here, we examine the effect of STC when it is applied with various other preprocessing steps such as motion correction (MC), motion parameter residualization (MPR), and spatial smoothing. Using 180 simulated and 30 real fMRI data, we quantitatively demonstrate that the optimal order in which STC should be applied depends on interleave parameters and motion level. We also demonstrate the benefit STC on sub-second-TR scans and for functional connectivity analysis. We conclude that STC is a critical part of the preprocessing pipeline that can be extremely beneficial for fMRI processing. However, its effectiveness interacts with other preprocessing steps and with other scan parameters and conditions which may obscure its significant importance in the fMRI processing pipeline.

## Introduction

Most functional magnetic resonance image (fMRI) scans are acquired using echo planar imaging (EPI), which rapidly acquires single or multiple 2D slices and stacks them to create a 3D volume. This process typically takes between 0.5 and 4 s (known as the repetition time, or TR), depending on the fMRI pulse sequence, field of view (FOV), and number of acquired slices (Stehling et al., 1991). Slices can be acquired sequentially or with interleave, in which a number of slices are skipped and acquired later during the TR. Interleaving allows more time for the partially excited spins in the adjacent slices to return to equilibrium, which attenuates the artifacts of radio frequency (RF) pulse excitation leakage. Regardless of the manner in which slices are acquired, they cannot be acquired instantaneously, and so the result is an accumulation of offset delays between the first slice and all remaining slices. In order for an accurate time series analysis to be carried out on the fMRI data, these temporal offsets between slices must be corrected for. Slice timing correction (STC) is the preprocessing step applied to correct for these slice-dependent delays, achieved by shifting the time series of each slice to temporally align all slices to a reference time-point.

In addition to STC, numerous preprocessing steps are required to prepare fMRI data for statistical analysis. Surprisingly, there exists no consensus on the order in which these preprocessing steps should occur, and the interaction of STC on motion parameter estimates has only recently been investigated (Cox, 1996; Power et al., 2017). It has been shown in previous studies that the order and the steps included in the preprocessing pipeline significantly affect the resulting statistical analysis (Zhang et al., 2009; Carp, 2012; Churchill et al., 2012, 2015). One previous report examined motion correction (MC), physiological noise correction, and motion parameter residualization (MPR) (Churchill et al., 2012), however, in this study, the effect of these steps was only evaluated based on their inclusion or omission from a fixed order in the pipeline, and STC was performed at the beginning of every pipeline. Thus, the contribution of STC on different steps in the pipeline remains unclear. To further contribute to this area of research, we examine whether the order of these steps will significantly impact resulting statistical parametric maps.

In a previous study, we directly compared the performance of different STC methods to each other (Parker et al., 2016). In the current study, we aimed to examine the interaction of these STC methods with other preprocessing steps. We have identified five areas common in fMRI analysis where the role of STC is largely uninvestigated: (1) The order of STC and MC in the preprocessing pipeline without spatial smoothing, (2) The order of STC and MC, and its interaction with MPR, without spatial smoothing, (3) The interaction of spatial smoothing and STC, (4) STC on data sampled with a fast TR, and (5) STC on functional connectivity analysis using independent component analysis (ICA). Using both real and simulated data, we quantitatively examined the effect of STC on data acquired with various levels of motion and slice acquisition sequences. We hypothesized that different combinations of motion and slice acquisition orders would alter the effectiveness of different preprocessing pipelines. We also use different STC techniques to see if the STC algorithm has any effect on pipeline order. We use FSL and SPM’s slice timing routines, where FSL implements a low-order Hanning windowed sinc interpolation, while SPM uses a frequency-domain phase shift. Finally, we apply a STC method developed by our group, FilterShift (FS), which implements a moderate-order Kaiser-windowed sinc function.

### Pipeline Order Without Spatial Smoothing

Some studies suggest that the optimal order of STC and other preprocessing steps such as spatial realignment depends on both the level of motion and the slice acquisition (Or interleave) order (Sladky et al., 2011). Motion may exacerbate the STC problem, introducing not only shifts in time but also in space. The effects of motion will change based on the slice acquisition orders. Different slice acquisition orders will result in visually different volumes, even if they undergo identical motion. This will lead to inaccurate and inconsistent motion parameter estimates. In these instances, it has been shown that STC before spatial realignment alters the motion parameter estimates and result in less accurate spatial realignment (Power et al., 2017). Likewise, as spatial realignment applies spatial transformations to the data, a voxel’s time series after spatial realignment may contain values interpolated from adjacent slices which were sampled at different offsets. Thus, applying a single time shift to a voxel containing data sampled with different delays is intuitively flawed. Therefore, it is important to investigate which order of the pipelines would be optimal in the preprocessing pipeline.

### Motion Parameter Residualization

Motion parameter residualization is the practice of including motion parameters in the GLM as nuisance regressors used for residualization which orthogonalizes the BOLD activity from the motion parameters. Because the motion parameters are typically estimated during the realignment step, the effectiveness of motion parameter residualization may change depending on the level of motion, the accuracy of the motion parameter estimation, and the order in which STC and MC are performed. The strategies for MPR vary greatly from study to study. Some groups only include the six rotation and translation parameter estimates, while others include any number of additional derivatives and quadratics in an attempt to capture any non-linear motion related signal fluctuations (Satterthwaite et al., 2013). There is still active research regarding the best kinds of covariates to include in the residualization, and the process is a common addition to modern preprocessing pipelines (Ciric et al., 2017). Regardless of the specific covariates used, the effectiveness of MPR relies on accurate motion parameter estimates. Given the interaction between subject motion and slice timing, it follows that the STC and MC preprocessing steps will have an impact on the motion estimates used for MPR, and indeed the MC itself, as it has been shown in the field (Power et al., 2017). Thus we investigate the effect of MPR on the effectiveness of STC in this paper.

### Spatial Smoothing on STC

Spatial smoothing is another preprocessing step that interacts with STC. Spatial smoothing has the effect of distributing a voxel’s intensity to any surrounding voxels that fall within the smoothing kernel, thus altering their time series. With a relatively small smoothing kernel, this may impact slices differently, depending on their delay and the type of interleave acquisition. Slices with large delay will have the most error, and will be averaged with adjacent slices with smaller delay. Depending on noise, motion, and slice acquisition order, this can attenuate some of the error in the large delay slice, and create a more accurate time series. Conversely, slices with small delays and errors may be averaged with adjacent slices with large delays and errors, reducing their accuracy. Because spatial smoothing operates across slices with different delays, it is important for us to investigate the interaction of STC, a slice-specific process, and spatial smoothing, a multi-slice process.

### STC on Short TRs

As new imaging techniques such as simultaneous multi-slice acquisition (SMS) become more and more common, acquisition times of whole volumes become faster (Barth et al., 2016). It is generally considered that with such short TR, STC is an unnecessary step with little value. This is based on the assumption that the BOLD signal is generally quite slow, and so any signal change due to temporal offsets is likely to also be small. However, the GLM is extremely sensitive to small shifts in signals, and to our knowledge no study has quantitatively demonstrated that STC is futile for short TR acquisitions. With the development of multiband EPI and improvements in the pulse sequence, whole brain volumes can be acquired in just fractions of a second (Feinberg et al., 2010). In our previous study, we showed on simulated data that traditional STC techniques showed little to no benefit with a fast TR, while our method still provided significant improvement (Parker et al., 2016). In this paper, we extend that analysis to real data acquired with a short TR. The Human Connectome Project (HCP) is one such study that employs this acquisition technique to achieve a TR of 0.72 s for task-based and resting-state fMRI. The HCP publishes an extensive pre-processing pipeline for all their data, which ignores STC due to the high sampling at which the volumes are acquired (Glasser et al., 2013). It is argued that the benefit from STC is insignificant at higher sampling rates. Because we have found no thorough investigations into this matter, it is important for us to examine the effect of STC on short-TR data.

### STC on Functional Connectivity Analysis

Functional connectivity (FC) analyses are becoming increasingly popular in the current literature (Sala-Llonch et al., 2015). In FC, it is not necessary to use an external regressor for a GLM analysis. There are many methods to extract inherent FC, such as seed based connectivity (Biswal et al., 1995a), ICA (Beckmann and Smith, 2004), and even graph theory (Buckner et al., 2009; Rubinov and Sporns, 2010). These methods rely only on the intrinsic fluctuations in each voxel, and its similarity to other voxels in the brain to extract functionally connected regions. Previous work has already examined the interaction of STC with resting state data connectivity, and have shown no significant effect (Wu et al., 2011). However, this study employed a 6mm spatial smoothing kernel to the data, which will greatly reduce the effectiveness of STC. It’s also important to consider that resting state fluctuations (in the default mode network, for example) are very slow, low frequency signals (<0.1 Hz) (Biswal et al., 1995b). It has already been shown that STC has little effect for low-frequency signals, such as block design tasks (Sladky et al., 2011), and we expect the same principle to carry over to resting state fMRI (rsfMRI). Functional connectivity analysis can be extracted from resting-state fMRI data as well as task-based fMRI data (Biswal et al., 1995a). In fact, recently task-based functional connectivity networks have gained much more popularity in the field (Di et al., 2013; Krienen et al., 2014; Stern et al., 2014). Our group recently demonstrated that one of the well-recognized functional connectivity networks, the default mode network, can be extracted from both task-based and resting-state fMRI data (Razlighi, 2018). Regardless of whether the scan is “task” or “resting,” the process of extracting independent components remains identical, however, given the relatively faster temporal characteristics of task-based BOLD signals (compared to resting-state BOLD signals), we expect STC to have a larger impact on FC when used on task-based fMRI. In our analysis, we used ICA to extract functional components. Because of the popularity of STC, and the missing investigation into task-based FC, we examine the effect of STC on functional connectivity analysis.

## Materials and Methods

### Simulated Data

It is difficult to evaluate the performance of preprocessing methods on real fMRI data because the true underlying BOLD signal is always unknown. Because of this, we created simulated datasets with a known, underlying BOLD signal for a quantitative comparison. Data was simulated using the same procedure described in Parker et al. (2016). Briefly, our simulated fMRI scans used real subject brain morphology by temporally averaging all volumes from a motion corrected real subject fMRI scan. We used the same subject’s structural segmentation from FreeSurfer (Fischl et al., 2002, 2004), and inter-modal rigid-body registration with FSL (Greve and Fischl, 2009) to obtain anatomical ROI masks in the fMRI space. We simulated neuronal activity consisting of sequences of 20 boxcar pulses with jittered onsets and randomly generated durations for each ROI. These stimuli were convolved with the canonical HRF to generate the hemodynamic response. Both HRF and neuronal stimuli were sampled at high frequency to facilitate in simulating motion and slice sampling. Cardiac and respiratory artifacts were simulated by adding a single sinusoid at *f*_c_ = 1.23 Hz for cardiac and another sinusoid at *f*_r_ = 0.25 Hz for respiratory noise. The magnitude of the cardiac artifacts is modulated based on the power of the cardiac artifact found in a real fMRI dataset. To do this, the Circle of Willis was manually identified, where the cardiac signal dominates the fMRI time-series. Next, the frequency range of the aliased cardiac signal is detected from the signal at the Circle of Willis. Then, the power spectrum of every voxel was examined in the detected frequency range, and the amplitude of the simulated physiological artifact is scaled with the power computed from the detected frequency range. Finally, a low thermal noise level was added to the simulation, consisting of white noise that made up 5% of the signal’s energy. The hemodynamic signal was scaled so that the standard deviation equals 4% of the mean signal’s magnitude, comparable to a robust signal in the visual cortex. This scaled signal was added to the mean value image. Because we know exactly where the signal is located, we can compare voxels directly from these regions without the need for spatial normalization.

We simultaneously simulated the effect of head motion and slice timing as well as their interaction. To do this, we use motion parameters estimated from real subject scans. We up-sampled these motion parameters from real subject movement inside the scanner (which includes rotations and translations along all three axes) by the number of slices in the simulated scans, using spline interpolation. For instance, if there were 40 slices in each volume, we up-sampled the motion parameters by 40 times the original fMRI sampling frequency (1/TR). We then transformed the brain volume 40 times according to the upsampled motion parameters between each pair of consecutively acquired fMRI volumes. This gave us the position of the brain volume at the instance of each slice acquisition. Then we sampled the slice from the transformed volumes according to the timing of each slice. The high temporal-resolution BOLD signal present in each voxel was also sampled according to the timing of each slice, which simulated slice timing, creating a lag or delay in sampling between slices. A more detailed description about this simulation can be found in Parker et al. (2016).

We generated 20 simulated scans from a single subject’s morphology. Each subject was given a unique BOLD time series for every region of the brain. For each time series, a simulated scan was created with three levels of motion: high, medium, and low. The motion levels were classified by a set of real subjects’ mean frame-wise displacement (mFWD) inside the scanner (Low: mFWD < 0.1 mm, med: 0.25mm < mFWD < 0.4 mm, high: 0.6 mm < mFWD < 0.7 mm) (Power et al., 2012). Simulated data with each motion level was then sampled with the three interleave schema; sequential (Interleave 1), even odd (Interleave 2), and every 6th (Interleave 6). Thus, each subject’s fMRI scan was simulated a total of nine times, each with unique interleave and motion parameters. Synthesized fMRI scans consisted of 10 min of scanning with an in-plane acquisition matrix of 112 × 112, and 37 slices. The voxel size was set to 2 mm × 2 mm × 3 mm. The TR was equal to 2 s for all experiments. Thus, with twenty BOLD time series each simulated under nine different conditions, we created 180 unique simulated fMRI scans.

### Real Data

We chose a task with high temporal sensitivity and a robust response for this study. Thirty right-handed healthy subjects (17/13 young/old; proportion female/male: 0.53/0.61, age mean ± std: 25.5/64.9 ± 2.4/2.2 years) were presented with event related visual (flashing checker boards) stimuli with random onset and duration while undergoing fMRI. To ensure attention to the stimuli, subjects responded with a button press at the conclusion of each visual stimulus. Functional images were acquired using a 3.0 Tesla Achieva Philips scanner with a field echo echo-planar imaging (FE-EPI) sequence [TE/TR = 20 ms/2000 ms; flip angle = 72°; 112 × 112 matrix size; in-plane voxel size = 2.0 mm × 2.0 mm; slice thickness = 3.0 mm (no gap); 41 transverse slices per volume, 6:1 Philips interleaved, in ascending order]. Participants were scanned for 5.5 min, with at least 37 events of visual and auditory stimuli. Subjects were stratified based on their mFWD over the entire scan period. Ten low motion (mFWD < 0.14 mm), ten medium motion (0.14 mm ≤ mFWD < 0.2mm), and ten high motion (mFWD ≥ 0.2 mm) subjects were selected for each group. We used this data for both GLM analysis and the model-free ICA analysis commonly used in FC analysis of fMRI data.

In addition, we used the “*100-unrelated*” dataset from the Human Connectome Project (HCP), which consisted of 100 healthy and young participants (age range = 22–35 years, m/f = 52/48) to evaluate the benefit of STC on data with short TR and multiband acquisition (Van Essen et al., 2013). HCP data were acquired on a customized Siemens 3T Skyra scanner with a multiband EPI sequence [TE/TR = 33.1ms/720 ms; flip angle = 52°; FOV = 208 × 180 mm; voxel size = 2.0 × 2.0 × 2.0 mm; 72 axial slices; multiband factor = 8]. We used the N-back working memory scan, and modeled all correct responses as an event-related regressor.

### fMRI Data Statistical Analysis

Assessing the effectiveness of STC methods is extremely challenging due to its interaction with motion (Kim et al., 1999), and the fact that its improvement is slice-dependent. The benefit of STC could appear absent if the majority of activation falls on a slice with low temporal delay due to the fact that very little temporal shifting is encountered in these slices. This is analogous to examining the effect of motion correction on data with extremely low motion. In most typical STC techniques, one slice is chosen as the “reference slice,” and all other slices are shifted temporally to match the time at which the reference slice was acquired. The effect we see due to STC is directly proportional to the size of this temporal shift. In fact, if the activation falls on the reference slice, then no shifting occurs, and we will see absolutely no difference between STC and uncorrected data. This could be mistaken as evidence that STC may not be required in the preprocessing pipeline. Furthermore, depending on an individual’s brain size, position and head orientation in scanner, the same brain region may fall on different slices with different acquisition delays from subject to subject. This makes it extremely difficult to compare STC methods on a given region across subjects, as shown in our previous publication (Parker et al., 2016). Therefore, it is important to evaluate the effectiveness of STC on slices with the maximal temporal delay across subjects. For all experiments in this study, we choose the first slice acquired at the beginning of the TR as our reference slice. Therefore, a “large delay slice” refers to a slice acquired toward the end of the TR, while a “small delay slice” refers to a slice acquired toward the beginning of the TR. This classification obviously depends on which slice is used for the reference slice.

Assuming all subjects are scanned with identical scan parameters, the easiest way to evaluate STC is to identify one high-delay slice in native space that intersects a region of activation in each subject. Higher-level cognitive tasks and contrast maps can sometimes result in only small regions of activation which vary spatially from subject to subject, making these kinds of studies inappropriate for investigating STC. Instead, a task with a well-known, robust, and spatially large activation should be used to ensure that every subject has some activation on a chosen high-delay slice. In our evaluation, we generated simulated data with the same subject brain morphology to control for brain shape differences. For real data, we used only the voxels in subjects’ native space that are located in slices with moderate offset delay and have significant activation for an attended flashing checkerboard visual sensory task.

Both simulated and real data underwent various preprocessing pipelines consisting of the following modules in different orders: rigid body spatial realignment (motion correction or MC) was applied with FSL (*mcflirt*, Jenkinson et al., 2002) using rigid-body registration of all volumes to the middle one. STC (temporal realignment) was performed using our in-house method FS, as well as the FSL and SPM default techniques. All STC methods temporally aligned the data to the first slice, acquired at the beginning of the TR. FS is described in our previous publication and summarized here (Parker et al., 2016). Our method relies on the Nyquist–Shannon sampling theorem, which states that a signal sampled at twice its Nyquist frequency can be optimally reconstructed by zero-padding and low pass filtering. Using this, as well as an understanding of digital filtering and the properties of our BOLD signal, we optimized the STC algorithm by upsampling the data, moderately increasing the filter order and addressing initialization artifacts with a circular padding scheme. Finally, an optimal window function is selected for our low pass filter (LPF) – a Kaiser windowed sinc, for its tradeoff between a stable pass band, steep transition width, and suppression of the stop band. The cutoff frequency is adjusted to remove high-frequency noise above 0.21 Hz, which is the highest frequency present in the canonical HRF. The upsampled data is then resampled at a desired offset, to correct for the slice timing artifact. As we have shown in our previous work (Parker et al., 2016), every interpolation scheme can be represented and implemented as low-pass filtering with a specific kernel. This might raise the question, why the proposed FS method (which is essentially another low-pass filter) outperforms the interpolation-based methods that were being used in FSL and SPM. While this has not been discussed extensively in the fMRI field, we need to emphasize that the filter type, order, cut-off frequency, window type, transition to pass-band ratio, zero-padding scheme, and most importantly its implementation are all extremely important factors to be considered in any digital filter design, and could substantially affect the performance of the filter. In that sense, one might consider FS theoretically as a modified implementation of the low-pass filter that is often used in fMRI pre-processing pipeline.

We also created a “gold standard” method to compare STC techniques by constructing a slice-dependent shifted regressor for each slice. These shifted regressors account for the slice dependent acquisition offset delay. This method only applied a LPF to the data, as most STC techniques inherently have a small amount of low pass filtering. For a reliable comparison, the same filter parameters used in our FS method were used for the Shifted Regressor (SR) technique. In theory, this method should produce the best results, if no 3D processing algorithm (e.g., 3D smoothing, or 3D spatial transformation) has been applied in the pre-processing, as those methods combine data from different slices with different offset delays, which would alter the signals of each voxel.

In order to focus purely on the effect of different STC methods, only the specified preprocessing steps are applied to the data. We developed a standard generalized linear model (GLM) in Python and used it to model observed fMRI data *Y* at each voxel as a linear combination of regressors *X* which were created by convolving the double gamma HRF with the stimulus timing function. We used a standard GLM model shown below:

Y=X⋅β+e

where β coefficients were obtained using the ordinary least square estimate and given by,

$$\beta ={\left(X^{T}\,X\right)}^{-1}\,X^{T}\,Y$$

Standard GLM statistical inference was performed to obtain the t-statistics and significance level of activation for each voxel independently. In our previous publication, we compared the t-statistics from the GLM directly. In this paper, we compare the standardized beta value, described as:

$$\beta_{s}=\beta \,\frac{\sigma_{x}}{\sigma_{y}}$$

Where σ_x_ is the standard deviation of the regressor X, σ_*y*_ is the standard deviation of the time series Y. If there is only one regressor in the matrix X, then this value is mathematically equivalent to the *Pearson* correlation coefficient of the regressor with the time series. The correlation provides a more intuitive measure that is normalized, and there for more comparable across different analyses. We assume that a pipeline is better if it is able to construct a time series that is more correlated to the regressor. While there are many methods for fMRI quality assessment, the *Pearson* correlation coefficient is a good choice for extracting the relationship between two variables (Tegeler et al., 1999; Miller et al., 2002; Wagner et al., 2005; Caceres et al., 2009). We then convert the correlation to a *z* score using Fisher’s-z transform for statistical comparison. We refer to this as the voxel’s *z*-score, and it is used as the basis of comparison throughout the paper.

### Voxel Selection

Only voxels in a slice with high acquisition delay in either real or simulated data were considered for this selection. For real data, we selected slice 17, (1.46 s delay from the beginning of the TR), and for simulated data we selected slice 18 (1.78 s delay from the beginning of the TR). Previous studies have demonstrated that the benefit of STC is directly proportional to the temporal offset from the selected reference slice, and have shown that parameter estimates have reduced variance from slice to slice, and remain unbiased after STC (Sladky et al., 2011). In a previous publication, we have shown that the difference between STC and uncorrected data is minimal in slices with low delay (Parker et al., 2016). This paper is primarily concerned with the effects of STC on fMRI data in different preprocessing pipelines. In order to evaluate this, we must examine voxels that are affected by the STC process. Just as the effects of MC can only be seen on volumes with motion present, the effects of STC can only be seen on slices with temporal delay. So the evaluation of the STC methods and their interaction with other processing steps would be meaningless if it is done on slices with minimal or no time delay, just as evaluation motion correction technique on scans without motion would give invalid results. Because of this, we focused only on high delay slices to examine the effect of STC and its interaction with preprocessing steps where it’s most detectable, rather than comparing the effect of STC across all slices.

We selected the left superior frontal (LSF) region as our region of interest (ROI) for simulated data since the superior frontal region is one of the few regions that spans over 20 slices, thus including slices with various acquisition delays. Therefore, we can guarantee that some part of the region will fall in a high-delay slice regardless of motion and subject morphology. It also spans from the center of the brain to the frontal region, which will capture many different kinds of motion artifacts, as the same motion may produce different, even opposite signal changes in different regions of the brain (Power et al., 2012). Only voxels in the LSF ROI were used in all simulated data analysis, and the known underlying BOLD signal assigned to the LSF ROI was used as our regressor.

**FIGURE 1:**
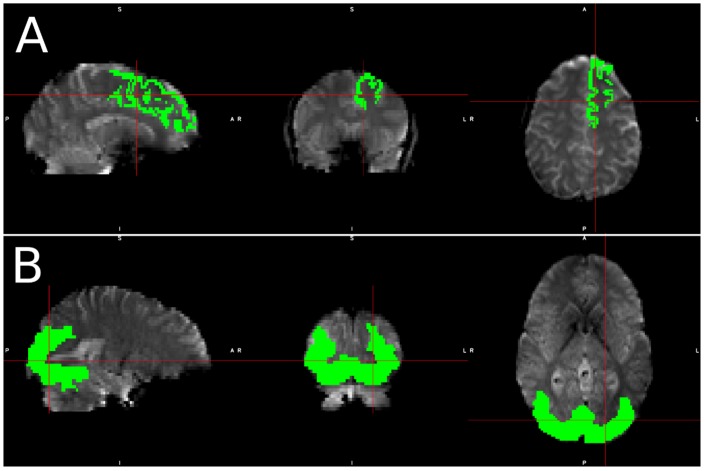
Visualization of spatial ROI’s used in analysis. Spatial maps for ROI’s used in **(A)** simulated data, which was identical for each simulated subject, and **(B)** Real data, in which the ROI varied slightly from subject to subject due to individual subject morphometry.

For real data, we created an ROI for each subject that included voxels where we expected significant visual activation. This ROI was created by transferring a group level activation mask for the visual stimulus back into each subject’s native space. This group level activation mask was created by running a full default FSL first level analysis, which consists of the following steps: (a) spatial realignment, (b) STC, (c) 3D smoothing with FWHM = 5 mm, (d) intensity normalization (e) temporal filtering (125 s cutoff) (f) GLM with prewhitening, and a second level analysis including: (a) spatial normalization, (b) full Bayesian linear model (Beckmann et al., 2003; Woolrich et al., 2004), and (c) cluster-wise multiple comparison correction (z threshold 2.3, cluster significance threshold *p* = 0.05). Only voxels in the activated regions were used in our real data analysis. Region masks for real and simulated data can be seen in Figure 1. The large ROI from the group level is only used to confine voxel selection to an area that is neurophysiologically task-relevant, to avoid false positives. For instance, we want to prevent selecting a false-positive voxel in the motor cortex for visual stimulation. One could just replace these ROIs with an anatomical mask of the visual cortex without having a significant alteration on the results. For real data, the average size of this region across all subject’s native space was 8704.3 voxels (104.6 cm^3^), with a standard deviation of 974.6 voxels. When confined to a slice of high delay, the average size of this mask was 514.0 voxels (6.2 cm^3^), with a standard deviation of 83.4 voxels. Despite the fact that this ROI was identified using a pipeline that performs MC before STC, the spatial smoothing used in the group level analysis makes the resulting mask large enough so that it will not bias the results to favor MC before STC pipelines. The large ROI is necessary, as individual differences in head position and brain function do not guarantee that all subjects will have significant activity in a single anatomical region if it’s too small. While it’s possible that this ROI may cover different anatomical regions over different subjects, the purpose of the group level analysis was to identify regions functionally and neuro-physiologically associated with the task. Since the function of the brain is what is associated with the BOLD signal, this allows us to identify the regions that are most likely to have the same underlying BOLD signal. This region spans over multiple slices, giving us a good chance at identifying significant voxels within the functional region, on a slice of high temporal delay.

**FIGURE 2:**
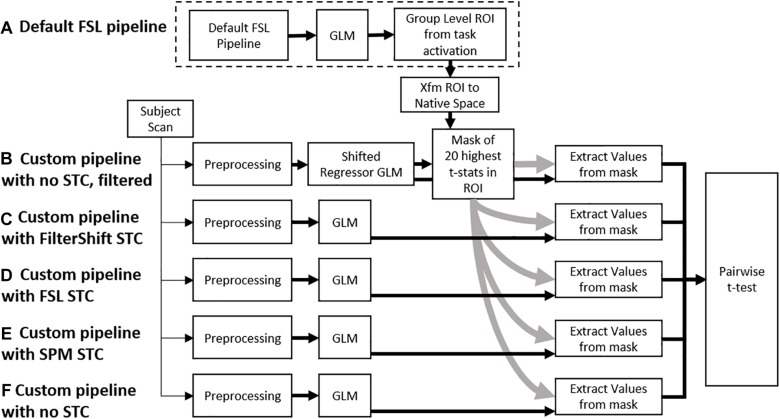
A flowchart of the processing pipeline for real data. **(A)** A default group level GLM is run on all subjects to obtain a region of activation, which is binarized into a mask and transformed back into each subject’s native space. This mask is used as an ROI to examine *t*-statistics from five different processing pipelines: **(B)** No STC, but the data is temporally lowpass filtered, and slice-dependent regressors shifted to account for the acquisition offset are used. **(C)** Our in house FS STC method is used. **(D)** FSL STC is used. **(E)** SPM STC is used. **(F)** No STC is used, and regressors are not shifted to account for slice offset. The statistics from pipeline b are used to identify 20 voxels with the highest t-statistics in the ROI. The standardized beta values from these 20 voxels are then extracted from the other pipelines, and the values are compared in a pairwise *t*-test.

Voxel selection was done exclusively from within the generated ROI mask and selected slice with high temporal acquisition delay. We used the SR technique to obtain the significantly activated voxels within the ROI without applying any spatial smoothing. In addition, the SR method does not require an extra STC step, thus making the choice of STC before or after re-alignment irrelevant. For any given subject, 20 voxels that had the highest t-statistics from the SR pipeline were identified. The *z*-score of the regressor to the time series of these 20 voxels were used to compare all STC methods and scan parameters. We then use a one tail pair-wise *t*-test on these values to compare the effectiveness of each pipeline. While we are using 30 fMRI scans from real and 20 from simulated data, each scan provided us with 20 voxels, each of which provides a unique fMRI measurement from different location in the brain. In other words, at each voxel location, we can establish an independent but similar comparison between the STC methods. Because of this, we consider each voxel a degree of freedom, giving us 199 DoF for real data (10 subjects for each motion level), and 399 for simulated data. The SR method theoretically does the best job identifying the regions that truly match the expected signal. By using the voxels with the highest statistics from the gold standard method, we identify the 20 voxels we are most certain are true positives, thus minimizing the possibility of type I error confounding our analysis, and allowing us to focus mostly on the effects of interpolation error from STC. It is important to note that these are not necessarily the 20 highest voxels for the other STC methods. Furthermore, from subject to subject, the location of these voxels is not constrained, as not every subject will exhibit the strongest response in the same location. Within each subject, we are comparing the same group of voxels across all different STC methods, so it is appropriate to use a repeated measures *t*-test to see if the preprocessing pipeline had any significant effect on the GLM, as done previously in the literature (Sladky et al., 2011). Our processing pipeline is illustrated in Figure 2.

The low number of voxels used in the comparison was chosen because certain real subjects only had 20 significant voxels present both in the region of interest, and also in the high-delay slice. The reason this number is low is because we do not perform spatial smoothing, which greatly increases the number of significant voxels, at the cost of a lower maximum *t*-value. In order to perform a repeated measures *t*-test, we need to compare values from the same voxels across multiple datasets. To ensure that the voxels we were comparing were in fact statistically significant in every comparison, we use the highest 20 voxels.

### Pipeline Order Without Smoothing

We created four different preprocessing pipelines to study the interaction of motion and the order of preprocessing steps. For simulated data, each slice acquisition order and motion level was processed with the following pipelines: (1) STC after motion correction (“After MC”). (2) STC before motion correction (“Before MC”). (3) STC only. (4) Motion correction only. (5) No correction. In this experiment, motion correction was carried out with FSL’s *mcflirt* spatial realignment tool using rigid body registration to the middle volume. For real data, subjects were divided into low, medium, and high motion groups, and the same five pipelines were run on each subject. For both simulated and real data, the top 20 voxels selected for statistical analysis in the respective ROI were identified using the “unsmoothed/SR” pipeline as the reference image. The LPF for the SR technique was applied before MC. For simulated data, we also extracted the HRF from these voxels using basic matrix deconvolution and compared it to the true HRF used to generate the simulation. We also computed the dice overlap of the location of each method’s top 20 voxels, compared to the top 20 voxels found using the SR method.

Prewhitening is an important step in the preprocessing pipeline. To assess the interaction between prewhitening and STC, we re-ran the real subject pipeline-order analysis with AFNI’s prewhitening routine, which is a voxelwise auto-regressive-moving-average ARMA(1,1) model, and compared the results to the non-prewhitened data. The interaction with STC has already been studied, and was found to be limited (Olszowy et al., 2019). To verify these results, we assessed this interaction with STC, both with and without MC.

We also use a common reliability measure to see if STC, and pipeline order, have any effect on the reliability of the data. For this experiment, we use the real, low-motion subjects to minimize confounds and focus on the effect of STC on reliability. We split each subject’s time series into two halves, and recomputed the GLM on each half with the appropriate segment of the regressor. A brain mask, created using FSL’s BET, is used to extract the beta values from the brain for each half of the time series. The correlation between the beta values from each half is then calculated. High correlation indicates good similarity between the analyses run on each half of the scan.

### Motion Parameter Residualization

We also examined the effect of pipeline order while including realignment parameter estimates as nuisance regressors, also known as MPR. Six motion parameters are estimated during the rigid body realignment stage described earlier, and included as nuisance regressors along with their temporal derivatives (rotation and translation for the x, y, and z axis) (Power et al., 2014). These parameters were acquired from FSL’s *mcflirt* spatial realignment tool, using rigid body registration to the middle volume. In cases with “No MC,” motion parameters were estimated after STC, but no realignment was done. Depending on the level of motion, more accurate motion parameters may be extracted before or after STC. This was run on a subset of 5 low, medium, and high motion subjects for both real and simulated data. As in the previous section, we used the “unsmoothed/SR” pipeline as the reference image for statistical analysis.

### Spatial Smoothing on STC

Slice timing correction is particularly sensitive to any sort of image processing/analysis that spans over multiple slices, the most common of such processes is 3D spatial smoothing. Smoothing is generally done to enhance the robustness of the statistical analysis results and reduce false positive rates (Mikl et al., 2008). Our goal in this experiment is to investigate the effect of 3D smoothing on the effectiveness of STC. Four different Gaussian non-linear smoothing kernels with FWHM = 3.5, 5, 6.5, and 8 *mm* were used to smooth simulated and real data using FSL’s smoothing method. Smoothing was applied by spatially convolving each 3D volume in the time-series with the appropriate 3D Gaussian kernel. For real data, the effect of smoothing was examined on high, medium, and low motion scans. For simulated data, low motion scans were used, and the effect of smoothing was examined for sequential, interleave 2, and interleave 6 acquisition methods. Each fMRI scan was first motion corrected and slice timing corrected before the various smoothing kernels were applied. We compared the performance of each STC method across the different smoothing kernels, where higher *z*-scores are considered better performance. For each motion/interleave case for real/simulated data respectively, the corresponding “unsmoothed/SR” image was used as the reference image for voxel selection.

### STC on Short TRs

To examine the benefit of STC on real data with high temporal resolution, we modified the HCP volumetric preprocessing pipeline described in Glasser et al. (2013) to output the results in native space, and removed any spatial smoothing to avoid the complications involved in assessing STC benefit, as described above. We refer to this custom pipeline as the “modified HCP pipeline.” We selected the N-back working memory task, described elsewhere (Barch et al., 2013), as our experimental data. As in the previous experiments, a default FSL first and second level analysis was run to obtain a group level activation mask of an event-related regressor modeling activation from all 2-back correct events. A python-based GLM was then run on each subject’s native space data with (a) the modified HCP pipeline with the gold standard SR, (b) the modified HCP pipeline with added FS STC, and (c) the modified HCP pipeline with no STC. We used the gold standard SR results as our reference image for statistical analysis.

### Effect of STC on Functional Connectivity Analysis

To examine the effect of STC on the extraction of functionally connected regions, we ran FSL’s MELODIC ICA analysis tool. MELODIC uses probabilistic ICA to extract spatially independent components along with their time series. MELODIC attempts to extract an optimal number of spatial/temporal components, so that when recombined they approximate the original data as closely as possible without overfitting (Smith et al., 2004).

For simulated data, we used low motion subjects with no added noise to attempt to minimize any confounds present in the analysis. We select data with a TR of 2 and interleave 6, which matches the parameters of our real data, for a more accurate comparison. Only STC was performed on the data, and no additional processing was done prior to running MELODIC ICA extraction. We manually identified a single IC which has the maximum spatial overlap with the task-related co-activation pattern found with the GLM in Section “Pipeline Order Without Smoothing,” and ran FSL’s dual regression to assess the statistical fit of this IC’s time series on the voxels present in the spatial IC (Beckmann et al., 2009). This provided us with z-statistics describing the significance of the IC’s time series for any given voxel. For this experiment, the z-statistics come directly from the statistical parametric map, rather than the *z*-score of a correlation as in the other analyses. Because there is no gold standard for ICA, we cannot use the same methods for comparing 20 voxels as described before. Instead, we simply extract significant z statistics generated from the dual regression that fall within a given ROI from both high and low delay slices. For real data, this ROI is the group level task activation map described earlier, and for simulated data this ROI is the LSF anatomical region where the signal was simulated in. We then examined the z-statistics within the mask separately for each slice, and together as a whole. We hypothesize that the uncorrected data will have lower z-statistics in high-delay slices, while STC data will have uniform z-statistic values over slices of all delays.

## Results

### Pipeline Order Without Spatial Smoothing

Table 1 shows the mean *z*-scores extracted from the spatial realignment/STC order experiment for simulated data described in Section “Voxel Selection.” These values are plotted in Supplementary Figure S1. The voxels with the top 20 t-statistics from the SR method are identified, and the *z*-scores are compared across different motion levels, interleave types, and STC methods. Within each method, the z-scores are extracted from three different pipelines: STC “before MC,” STC “after MC,” and only STC (“no MC”). The “Uncorrected” condition refers to scans with no STC, and only “MC” or “No MC,” where “No MC” refers to an unprocessed scan.

**TABLE 1:** Effect of the order in which STC and MC are applied in the preprocessing pipeline in simulated data.

	**Sequential**			**Interleave 2**			**Interleave 6**		
	***Low motion***	***Medium motion***	***High motion***	***Low motion***	***Medium motion***	***High motion***	***Low motion***	***Medium motion***	***High motion***
**FilterSift**									
*Before MC*	1.50 ± 0.24	0.62 ± 0.15	0.39 ± 0.14	1.50 ± 0.24	0.62 ± 0.15	0.39 ± 0.15	1.51 ± 0.24	0.62 ± 0.15	0.39 ± 0.14
*After MC*	**1.57** ± **0.27^‡^**	**0.64** ± **0.19**	**0.61** ± **0.17^‡^**	**1.56** ± **0.26^‡^**	**0.63** ± **0.18**	**0.56** ± **0.16^‡^**	**1.55** ± **0.26^‡^**	**0.63** ± **0.19**	**0.56** ± **0.17^‡^**
*No MC*	1.20 ± 0.26^‡^	0.38 ± 0.17^‡^	0.15 ± 0.14	1.20 ± 0.26^‡^	0.38 ± 0.17^‡^	0.16 ± 0.14^‡^	1.21 ± 0.26^‡^	0.38 ± 0.17^‡^	0.16 ± 0.13^‡^
**FSL**									
*Before MC*	1.52 ± 0.24	0.64 ± 0.19	0.57 ± 0.20	1.23 ± 0.14	0.60 ± 0.16	0.55 ± 0.18	**1.06** ± **0.11**	**0.59** ± **0.15**	**0.54** ± **0.17**
*After MC*	1.52 ± 0.24	0.64 ± 0.18	**0.60** ± **0.17**	**1.24** ± **0.15^∗^**	0.60 ± 0.16	**0.54** ± **0.15**	1.04 ± 0.11^‡^	0.58 ± 0.14	0.51 ± 0.14
*No MC*	1.18 ± 0.24^‡^	0.38 ± 0.17^‡^	0.15 ± 0.14^‡^	1.03 ± 0.17^‡^	0.37 ± 0.16^‡^	0.16 ± 0.14^‡^	0.92 ± 0.14^‡^	0.36 ± 0.15^‡^	0.16 ± 0.13^‡^
**SPM**									
*Before MC*	**1.18** ± **0.13**	0.60 ± 0.16	0.53 ± 0.18	1.11 ± 0.12	0.58 ± 0.15	**0.54** ± **0.17**	**1.05** ± **0.11**	**0.58** ± **0.15**	**0.53** ± **0.17**
*After MC*	1.17 ± 0.13	**0.60** ± **0.15**	**0.56** ± **0.15**	1.11 ± 0.12	0.58 ± 0.14	0.52 ± 0.14	1.03 ± 0.11^‡^	0.57 ± 0.14^∗^	0.51 ± 0.14
*No MC*	0.98 ± 0.16^‡^	0.36 ± 0.16^‡^	0.14 ± 0.14^‡^	0.95 ± 0.15^‡^	0.36 ± 0.15^‡^	0.15 ± 0.13^‡^	0.91 ± 0.13^‡^	0.36 ± 0.15^‡^	0.16 ± 0.13^‡^
**Uncorrected**									
*MC*	**0.95** ± **0.08**	**0.54** ± **0.12**	**0.52** ± **0.13**	**0.85** ± **0.08**	**0.51** ± **0.12**	**0.50** ± **0.12**	**0.77** ± **0.07**	**0.47** ± **0.11**	**0.47** ± **0.12**
*No MC*	0.84 ± 0.11^‡^	0.34 ± 0.14^‡^	0.14 ± 0.14^‡^	0.76 ± 0.10^‡^	0.32 ± 0.13^‡^	0.15 ± 0.13^‡^	0.70 ± 0.09^‡^	0.31 ± 0.12^‡^	0.15 ± 0.12^‡^

*Mean *z*-score of top 20 voxels identified in the Shifted Regressor “Before MC” case, extracted across various preprocessing pipelines for five simulated subjects. For the Shifted Regressor data, “Before MC” refers to when the low pass filter was applied to the data in the preprocessing pipeline. Pairwise *t*-tests were used to determine significant differences between pipelines for a given STC technique, relative to the reference case highlighted in gray (STC before MC). Significant differences are indicated with a “ ^∗^” for *p* < 0.01, and a “ ^‡^” *p* < 0.001. Bold numbers indicate the highest mean *z*-score of the three pipelines for a given interleave, motion level, and STC technique. Highlighted rows indicate the data used as reference in the *t*-test.*

From this table, we see that the results vary based on the level of motion, the slice acquisition orders, and the methods of STC. Significant differences are indicated with a “ ^∗^” for *p* < 0.01, and a “ ^‡^” for *p* < 0.001. All *t*-tests were calculated with respect to the STC “Before MC” case within each method/motion/interleave block, which is highlighted in gray, and the pipeline with the highest average *z*-score value is bolded within each STC/Interleave/Motion category. For FS, STC “After MC” significantly outperformed “before MC” across all interleave values for both low and high motion (*p* < 0.001), but not for medium motion, where there were no significant differences for the order of STC and MC. In all cases, both “After MC” and “before MC” outperformed “No MC.”

For FSL, pipeline order had no effect in sequential data. For interleave 2, “After MC” is significantly higher than “before MC” for low motion data (*p* < 0.01), but not for medium and high motion subjects. For interleave 6, we now see that “before MC” performs significantly better than “After MC” for low motion subjects (*p* < 0.001). For medium and high motion subjects, “before MC” does have a higher mean z-score than “After MC,” but it is not statistically significant (*p* > 0.01).

For the SPM corrected data, we see many similar trends. Pipeline order is largely irrelevant for both sequential and interleave 2 acquired data, with no significant differences between “After MC’ and “Before MC.” For interleave 6, we see that “Before MC” performs significantly better than “After MC” for low motion (*p* < 0.001), and for medium motion (*p* < 0.01), but not for high motion. Finally, for uncorrected data, “MC” always performed significantly better than “No MC” for all data sets (*p* < 0.001).

Interestingly, the effects of STC and spatial realignment seem to add non-linearly. For example, in the medium motion case with interleave 6, the application of MC to the data without STC raises the average *z*-score from 0.31 to 0.47 (52% increase). The addition of FSL’s STC without MC raises the *z*-score from 0.31 to 0.36 (16% increase). However, the addition of both MC and FSL’s STC raises the *z*-score from 0.31 to 0.58 (87% increase). This is greater than the summation of just MC or just STC. This highlights the importance of applying both STC and MC to fMRI data in the pre-processing stream.

**FIGURE 3:**
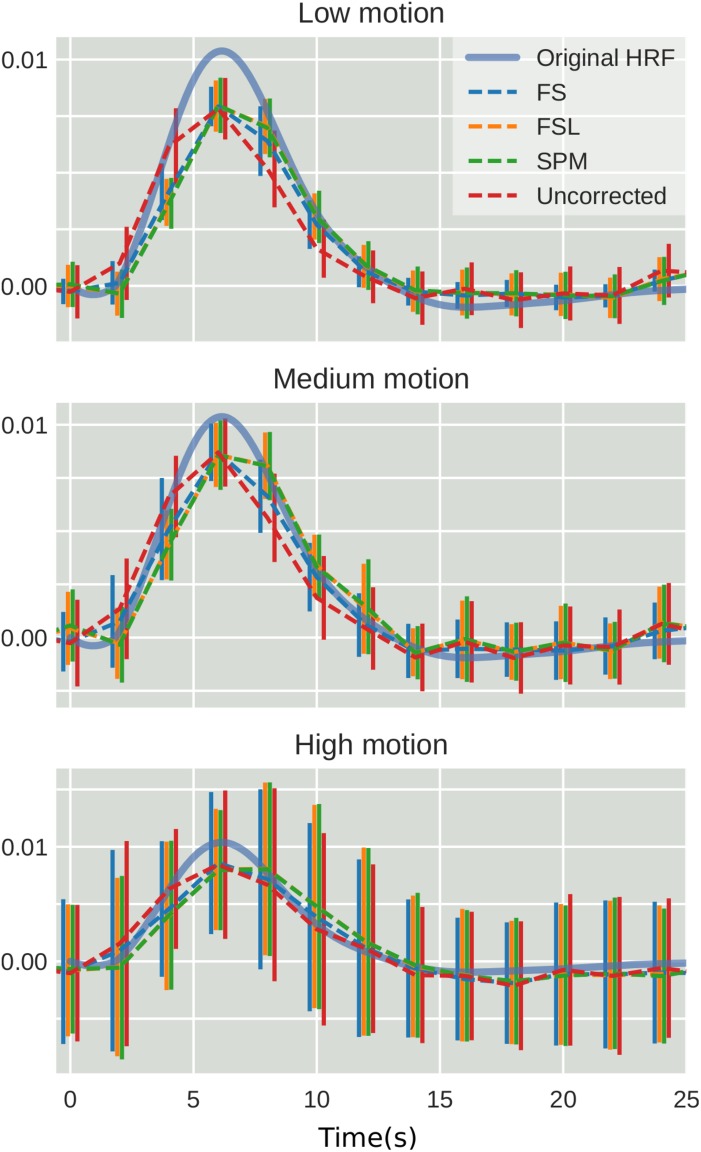
The effect of STC on the accuracy of BOLD response. Normalized HRF’s extracted from the top 20 voxels identified using the shifted regressor method for low, medium, and high motion in simulated data. HRF’s were extracted using FIR deconvolution for each STC method (dashed lines). The reference HRF used in the simulation is plotted as a solid blue line. For each motion level and STC method, HRF’s are combined across all pipeline orders.

**FIGURE 4:**
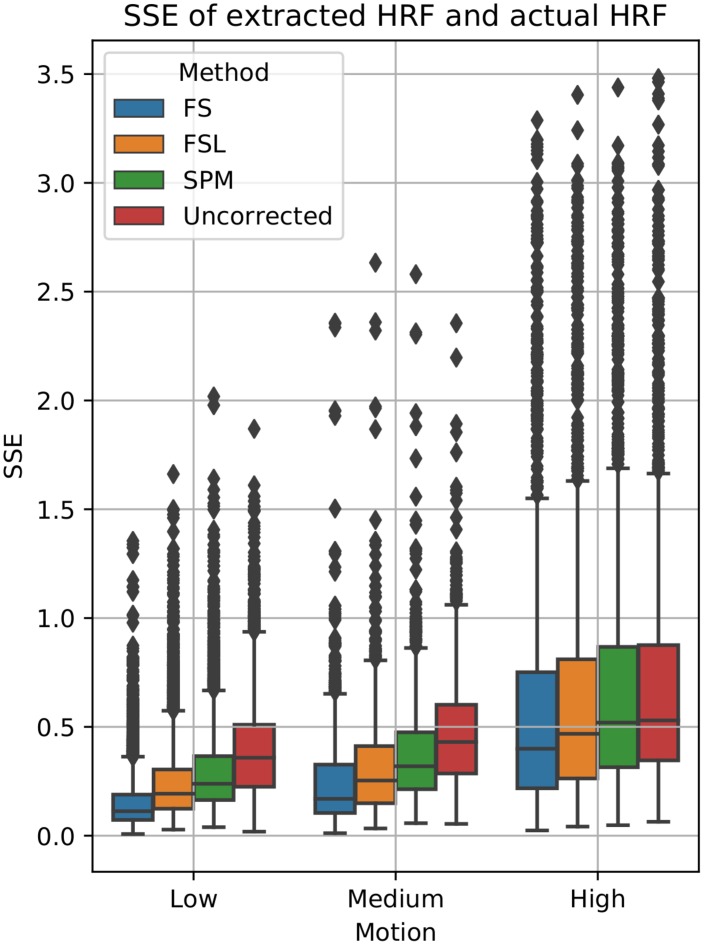
Quantification of BOLD HRF extraction error. SSE of normalized HRF’s deconvolved from STC data, compared to the canonical HRF used in the simulation for low, medium, and high motion. For each motion level and STC method, HRF SSE’s are combined across all pipeline orders.

To examine the effect of STC on estimated BOLD response parameters, the HRF was extracted using FIR deconvolution (Glover, 1999) from the 20 voxels identified in the previous analysis for simulated data, using the “STC after MC” pipeline, as it performed best for simulated data. A more accurate HRF indicates that the time series better matches the expected stimulus response. We computed the sum of squared error (SSE) between the extracted HRF and the true underlying HRF, both normalized. The HRF was normalized because each voxel was assigned a BOLD signal that was 4% of the mean intensity of that voxel. Thus, for every voxel, the magnitude of the extracted HRF should be different, meaning that absolute error does not fully describe the goodness of fit, as the size of the original signal must be accounted for. The extracted HRFs can be seen in Figure 3. For each STC method, the HRFs from all pipelines are included in the plots. It can be seen that the error of the estimates increases as motion increases. The SSE are visualized separately using a box-plot in Figure 4 for each of the STC techniques. For low motion, FS has a mean SSE of 0.15, while FSL, SPM, and uncorrected data have a mean of 0.24, 0.29, and 0.39 respectively. The error increases for medium and high motion, reaching a mean of 0.62 for FS, 0.67 for FSL, 0.72 for SPM, and 0.73 for uncorrected data in the high motion case. As shown, increasing the level of head motion increases the SSE and reduces the difference between the utilized STC techniques. Note, the line in the boxplot indicates the median.

**FIGURE 5:**
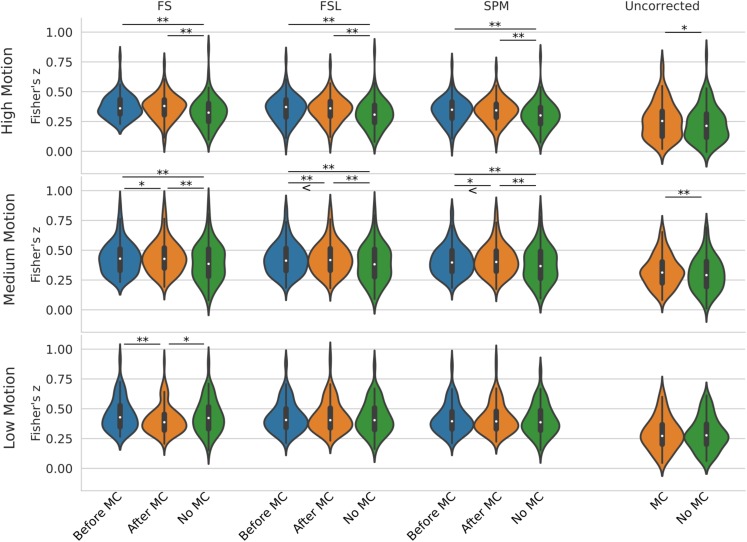
Effect of the order in which STC and MC are applied in the preprocessing pipeline in real data. *z*-scores from the top 20 voxels from a visual ROI of 30 real subjects’ time series using different preprocessing pipelines, with no smoothing. FS, FSL, and SPM slice timing correction were applied before motion correction, after motion correction, or without any motion correction. For the “Uncorrected” case, only motion correction was applied in the preprocessing pipeline. ‘<’ and ‘>’ Symbols indicate which mean is greater for cases that are not easily distinguishable.

Figure 5 shows a violin plot of real data and the effect of STC/MC order. For low motion, the only significant difference was found with the FS STC method, where STC after MC created significantly lower *z*-scores than STC before MC (*p* < 0.001), and STC only (*p* < 0.01). For all other STC methods in low motion subjects, there were no significant differences between any other pipelines, even for uncorrected data, which compares MC in the absence of STC. For medium motion, pipeline order had a significant impact for all STC techniques. For FS, FSL, and SPM, STC and MC together in any order performed significantly better than No MC (*p* < 0.001), and STC before MC performed worse than STC after MC (*p* < 0.01 for FS and SPM, *p* < 0.001 for FSL). In the medium motion case, MC significantly improved uncorrected data (*p* < 0.001). For high motion subjects, there is again no significant difference between pipelines applying STC before or after MC. However, the presence of MC again improves *z*-scores significantly across all methods (*p* < 0.001). For uncorrected data, MC again proved significantly beneficial (*p* < 0.01). Generally, for subjects with high and low motion, the order of the preprocessing pipeline seem to have no effect, while for the medium motion cases, MC before STC shows small but statistically significant improvement over STC before MC for FSL and SPM. Real data also highlights the importance of performing both MC and STC. For medium and high motion subjects, STC and MC performed together in *any* order (“Before MC” and “After MC” columns) always results in higher statistics than just STC alone (“No MC” column, *p* < 0.001).

**FIGURE 6:**
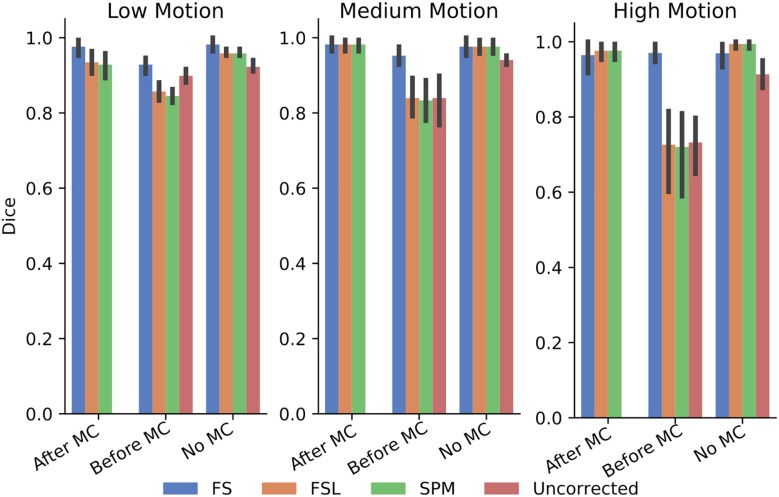
The similarity of the selected voxels in our evaluations of simulated data. Dice overlap in simulated data of top 20 voxels for each STC in various pipelines, compared to the top 20 voxels of the shifted regressor method within that pipeline. Uncorrected data in the “No MC” column refers to data that has had no MC or STC.

**FIGURE 7:**
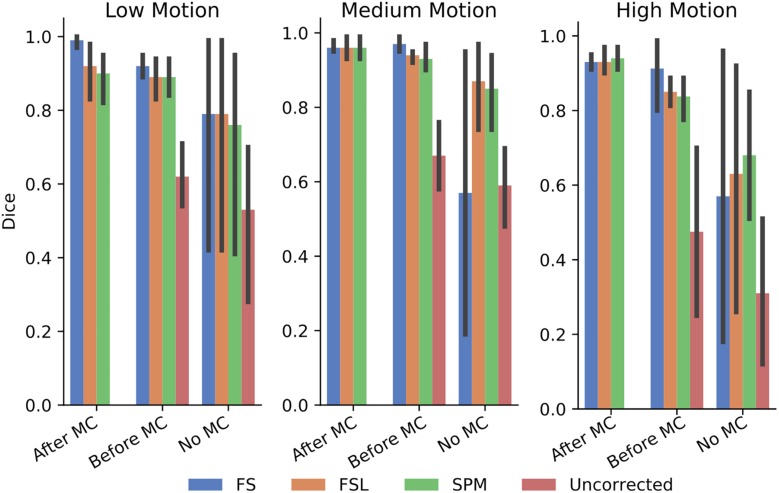
The similarity of the selected voxels in our evaluations of real data, Dice overlap in real data of top 20 voxels for each STC in various pipelines, compared to the top 20 voxels of the shifted regressor method within that pipeline. Uncorrected data in the “No MC” column refers to data that has had no MC or STC.

In order to further probe these results, we calculated the dice overlap of the top 20 voxels from each method in the high delay slice, compared to the top 20 voxels identified using the SR method. This was done for real and simulated data, and for each different pre-processing pipeline. Figure 6 shows the resulting Dice overlap coefficient of simulated data, and Figure 7 shows the same for real data. As shown, applying MC after STC (the “Before MC” column) typically lowers the dice overlap of the highest voxels relative to those of the “gold standard” method, which worsens as motion increases. In real data, we see a similar trend, with slightly lower dice overlap when MC is applied after STC. FS method has a lower dice overlap than other STC methods in real data for medium and high motion cases when MC is not applied.

To compare the interaction of STC and PW, we examine the *z*-scores of the pipeline with STC only, with and without PW. For low motion subjects, prewhitening did not have a significant effect on the *z*-scores for STC data (*p* > 0.05). The t-statistic maps from the prewhitened data had a mean correlation coefficient of 0.90 with the t-statistics maps from the non-prewhitened data across all subjects, while beta maps had a mean correlation coefficient of 0.83. All *z*-score averages from the prewhitened data were within 0.04 units of the corresponding value in the non-prewhitened data. This agrees with the literature in the field, that has found only small interactions between STC and prewhitening (Olszowy et al., 2019). Increasing the motion level, or including MC into the pipeline introduced more differences in *z*-scores between prewhitened and non-prewhitened data, indicating a possible interaction with prewhitening and motion. This relationship is beyond the scope of this manuscript, and can be addressed in future work.

To examine the effect of STC on reliability, the correlations of the low motion subjects’ beta maps are shown in Supplementary Figure S2. When examining the reliability, we found no significant differences between pipeline orders within each STC method with a two sample *t*-test (*p* > 0.1). We also ran a repeated measures t-test on the uncorrected data, and the STC only data for each method, which also showed no significant differences (*p* > 0.1). This shows that STC does not lower the reliability of the fMRI data.

### Motion Parameter Residualization

Residualizing motion parameters affected the results from the section “Pipeline Order Without Spatial Smoothing,” as shown in Table 2. Table 2 shows the summarized statistics for simulated data, and these results are plotted in Supplementary Figure S3. It is clear from Table 2 that the order of STC and MC are greatly affected by MPR. For all STC methods, across all levels of motion and all interleave methods, STC after MC provided the best *z*-scores (*p* < 0.001). For uncorrected data, MC only proved significantly better than no MC for low motion sequential data (*p* < 0.01), and high motion data for all interleaves (*p* < 0.01).

**TABLE 2:** Effect of the order in which STC and MC are applied in the preprocessing pipeline in simulated data, with MPR.

	**Sequential**			**Interleave 2**			**Interleave 6**		
	***Low motion***	***Medium motion***	***High motion***	***Low motion***	***Medium motion***	***High motion***	***Low motion***	***Medium motion***	***High motion***
**FilterShift**									
*Before MC*	1.00 ± 0.44	0.39 ± 0.25	0.14 ± 0.09	0.94 ± 0.41	0.39 ± 0.26	0.14 ± 0.09	0.78 ± 0.31	0.35 ± 0.22	0.13 ± 0.09
*After MC*	**1.33** ± **0.47^‡^**	**0.65** ± **0.37^‡^**	**0.50** ± **0.18^‡^**	**1.25** ± **0.47^‡^**	**0.63** ± **0.36^‡^**	**0.48** ± **0.17^‡^**	**0.97** ± **0.30^‡^**	**0.56** ± **0.28^‡^**	**0.43** ± **0.15^‡^**
*No MC*	1.01 ± 0.44^∗^	0.39 ± 0.25	0.14 ± 0.11	0.95 ± 0.41^∗^	0.39 ± 0.26	0.13 ± 0.11	0.78 ± 0.31	0.36 ± 0.22	0.12 ± 0.10
**FSL**									
*Before MC*	1.00 ± 0.43	0.39 ± 0.24	0.15 ± 0.10	0.87 ± 0.34	0.38 ± 0.22	0.14 ± 0.09	0.79 ± 0.29	0.35 ± 0.18	0.14 ± 0.09
*After MC*	**1.31** ± **0.46^‡^**	**0.65** ± **0.35^‡^**	**0.50** ± **0.18^‡^**	**1.08** ± **0.33^‡^**	**0.58** ± **0.25^‡^**	**0.47** ± **0.16^‡^**	**0.94** ± **0.25^‡^**	**0.53** ± **0.19^‡^**	**0.43** ± **0.14^‡^**
*No MC*	1.01 ± 0.44	0.39 ± 0.25	0.14 ± 0.11	0.87 ± 0.34	0.38 ± 0.21	0.14 ± 0.11	0.79 ± 0.29	0.36 ± 0.18	0.14 ± 0.10
**SPM**									
*Before MC*	0.84 ± 0.32	0.35 ± 0.19	0.14 ± 0.09	0.81 ± 0.30	0.37 ± 0.21	0.14 ± 0.09	0.78 ± 0.29	0.35 ± 0.18	0.14 ± 0.09
*After MC*	**1.03** ± **0.30^‡^**	**0.56** ± **0.24^‡^**	**0.47** ± **0.16^‡^**	**0.97** ± **0.27^‡^**	**0.54** ± **0.21^‡^**	**0.45** ± **0.15^‡^**	**0.93** ± **0.24^‡^**	**0.52** ± **0.19^‡^**	**0.43** ± **0.14^‡^**
*No MC*	0.75 ± 0.46^∗^	0.35 ± 0.19	0.14 ± 0.11	0.81 ± 0.30	0.36 ± 0.19	0.14 ± 0.10	0.78 ± 0.28	0.35 ± 0.18	0.13 ± 0.10
**Uncorrected**									
*MC*	**0.74** ± **0.27**	0.33 ± 0.16	**0.14** ± **0.09**	0.65 ± 0.23	0.31 ± 0.16	**0.14** ± **0.08**	0.59 ± 0.20	0.29 ± 0.14	**0.13** ± **0.08**
*No MC*	0.63 ± 0.38^‡^	0.33 ± 0.16	0.13 ± 0.10	0.65 ± 0.23	0.31 ± 0.16	0.12 ± 0.09^∗^	0.59 ± 0.20	0.29 ± 0.14^∗^	0.11 ± 0.09^‡^

*Mean *z*-score of top 20 voxels identified in the Shifted Regressor “Before MC” case, extracted across various preprocessing pipelines for five simulated subjects with motion parameters included as nuisance regressors in the GLM. For the Shifted Regressor data, “Before MC” refers to when the low pass filter was applied to the data in the preprocessing pipeline. Pairwise *t*-tests were used to determine significant differences between pipelines for a given STC technique, relative to the reference case highlighted in gray (STC before MC). Significant differences are indicated with a “ ^∗^” for *p* < 0.01, and a “ ^‡^” *p* < 0.001. Bold numbers indicate the highest mean *z*-score of the three pipelines for a given interleave, motion level, and STC technique. Highlighted rows indicate the data used as reference in the *t*-test.*

For real data, the effect of including motion parameters was small, and seemed to change based on motion level. These values are plotted in Supplementary Figure S4. For low and medium motion, MPR reduced the average *z*-scores across all methods by 0.03 and 0.04 respectively. For high motion, the average *z*-scores decreased by only 0.003. For low motion, the effect of combining STC and MC is enhanced, as STC “Before MC” is now significantly better than “No MC” for FS, FSL, and SPM (*p* < 0.001). For SPM, STC “After MC” also became significantly different from no MC (*p* < 0.01). For medium motion, the significant differences in pipeline order disappear for FSL and SPM. For high motion, the same significant differences present in the data without MPR were also found in this data set. Additionally, for FS, the pipeline order now significantly effected *z*-scores, with STC after MC resulting in higher *z*-scores (*p* < 0.01).

### Spatial Smoothing on STC

**FIGURE 8:**
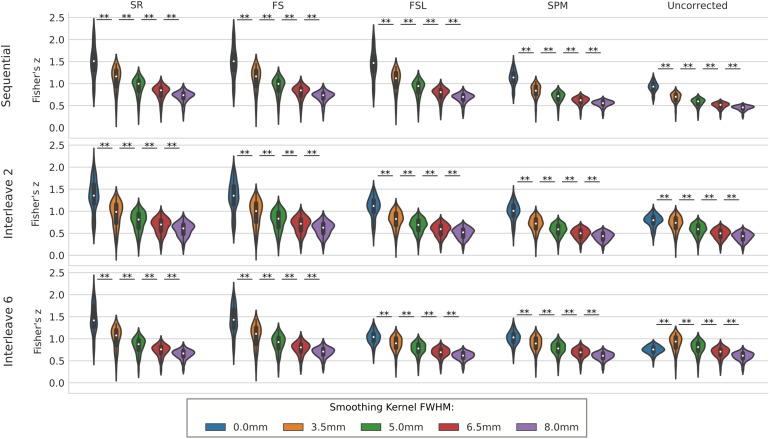
The effect of spatial smoothing on simulated data with STC. Violin plots show the resulting *z*-scores from the voxels with the top 20 t-statistics for 20 simulated data sets processed with smoothing kernels of 0, 3.5, 5, 6.5, and 8 mm. We examined the effect of smoothing for sequential acquisition, even-odd (interleave 2) and every 6th (interleave 6) acquisition (rows). For each interleave/smoothing condition, statistics were compared across 5 STC conditions: Shifted Regressor, FS, FSL, and SPM STC methods were examined, as well as uncorrected data. For each interleave, the voxels with the top 20 t-statistics were identified from the unsmoothed Shifted Regressor case. The *z*-score of these 20 voxels are plotted for all other STC methods and all other smoothing conditions. The effect of smoothing is seen as a lowering of the mean *z*-scores, as well as reducing the variance across STC methods due to the distribution of slice-dependent errors. For interleaved data, the Shifted Regressor method, which is supposed to be a gold standard, performs worse than our proposed FS method due to these distributed errors.

We compared the effect of spatial smoothing on STC using both real and simulated data. Figure 8 shows the results across simulated subjects with low motion for various types of interleave acquisition. For simulated data, increasing the kernel size reduces the mean *z*-scores monotonically as kernel size increases for all STC data under all interleave scenarios. For all interleave parameters and STC methods, each level of smoothing was significantly different from the following level (*p* < 0.001). Interestingly, mean *z*-scores from uncorrected data only decreased monotonically for sequential acquisition and interleave 2 acquisition. For uncorrected data with interleave 6, the mean *z*-score is 19% higher than the unsmoothed case at FWHB, and 6% higher than the unsmoothed case at FWHM of 5 mm. Beyond 5 mm, the smoothed uncorrected data no longer performs better than the unsmoothed data, dropping monotonically as the FWHM increases. It is also important to note the effect of smoothing on the SR method. For interleave 2 and 6, the average *z*-score for SR was lower than the average *z*-score for FS in all smoothed datasets. This effect is amplified as kernel size increases, and as interleave parameter increases. In other words, SR method can only be considered as a gold standard technique when there is no spatial smoothing applied, and any smoothing with kernel size larger than 3.5 mm will deteriorate its performance to the level where other methods could outperform it.

**FIGURE 9:**
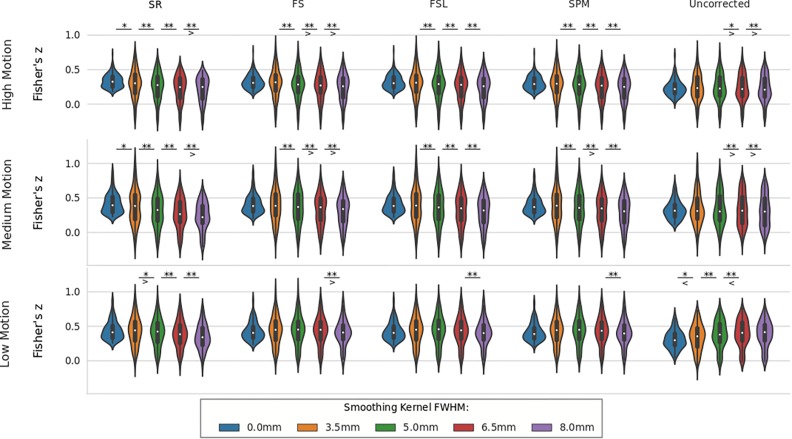
The effect of spatial smoothing on real data with STC. Violin plots show the resulting *z*-scores from the voxels with the top 20 t-statistics for 30 real data sets (10 low motion, 10 medium motion, 10 high motion) processed with smoothing kernels of 0, 3.5, 5, 6.5, and 8 mm. We examined the effect of smoothing for low, medium, and high motion levels (rows). For each motion/smoothing condition, statistics were compared across 5 STC conditions: Shifted Regressor, FS, FSL, and SPM STC methods were examined, as well as uncorrected data. For each motion level, the voxels with the top 20 t-statistics were identified from the unsmoothed Shifted Regressor case. The *z*-score of these 20 voxels are plotted for all other STC methods and all other smoothing conditions. The effect of smoothing is seen as a lowering of the mean *z*-scores, as well as reducing the variance across STC methods due to the distribution of slice-dependent errors. In real data, the “Gold standard” (Shifted Regressor) is outperformed by all other STC methods when large smoothing kernels are used. ‘<’ and ‘>’ Symbols indicate which mean is greater for cases that are not easily distinguishable.

For real data, the effect of spatial smoothing on the time series z-scores are shown in Figure 9. For low motion, FS, FSL, and SPM do not significantly change from the unsmoothed case compared to any other kernel size. The only significant differences between consecutive smoothing kernels are found between 6 and 8 mm for FS, FSL, and SPM. The SR method, on the other hand, has significantly lower z-scores between smoothing kernels 3.5 and 5 mm (*p* < 0.01). The mean *z*-scores continue to decrease significantly with kernel size 6.5 and 8 mm (*p* < 0.001). For uncorrected data, we see the same phenomenon from the simulated data, where the smoothed *z*-scores are significantly larger as the kernel size increases from 3.5 to 5 mm (*p* < 0.01), from 5 to 6.5 mm (*p* < 0.001), and from 6.5 to 8 mm (*p* < 0.001). This increase in *z*-score of the uncorrected data was expected, since smoothing blends the high delay voxels with low delay ones, which essentially performs similar to linear interpolation. For medium and high motion subjects, we see the same general decreasing trend in *z*-scores as larger smoothing kernels are applied to STC data. We no longer see the same initial increase in *z*-score in the uncorrected data. For all STC methods, the unsmoothed data has significantly higher *z*-scores than the smoothed data for kernel sizes greater than or equal to 5 mm (*p* < 0.001). For the SR method, this difference becomes significant at even just 3.5 mm (*p* < 0.01). For low and medium motion cases, the average *z*-score for SR data smoothed with an 8 mm kernel is even lower than any other STC method, and in fact even lower than uncorrected data in the medium motion case.

### STC on Short TRs

**FIGURE 10:**
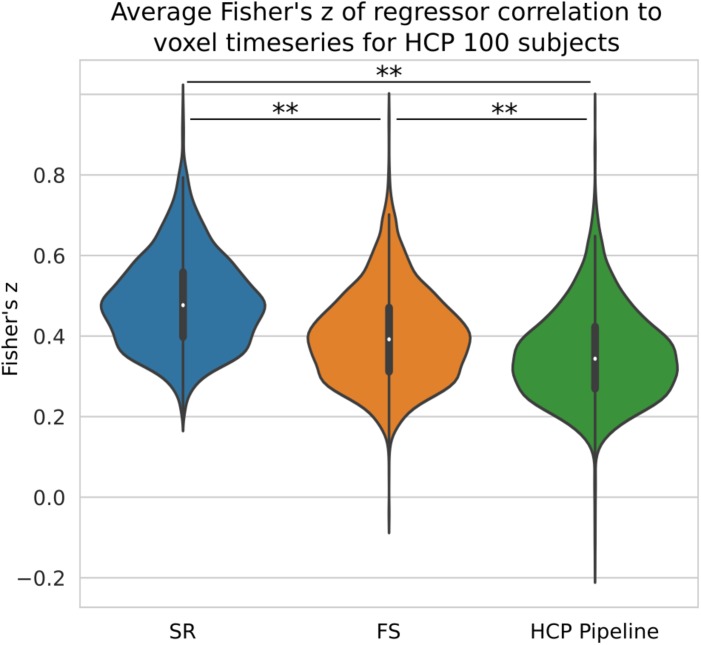
The Benefit of STC on short-TR data. *Z*-scores from voxels with the top 20 t-statistics for 100 HCP unrelated subjects. Violin plots show the combined *z*-scores of all 100 subjects from the 20 voxels with the highest *t*-values in the Shifted Regressor case when processed with the default volumetric HCP pipeline, with our proposed FS method, and with the Shifted Regressor. This shows the clear benefit of applying STC to short TR (0.72 s), multiband data.

We compared the *z*-score in the native space generated from HCP data processed with three different pipelines with no spatial smoothing. We have shown previously on simulated data that short TR data benefits greatly from the ideal LPF we use in FS. When performed on HCP data, we found our STC method significantly increased the mean *z*-score in the top 20 voxels by 13% (*p* < 0.001), shown in Figure 10. The SR method was further able to increase the *z*-score by 37% from the HCP default pipeline, further highlighting the sensitivity of low TR data to small temporal shifts.

### STC on Functional Connectivity Analysis

**FIGURE 11:**
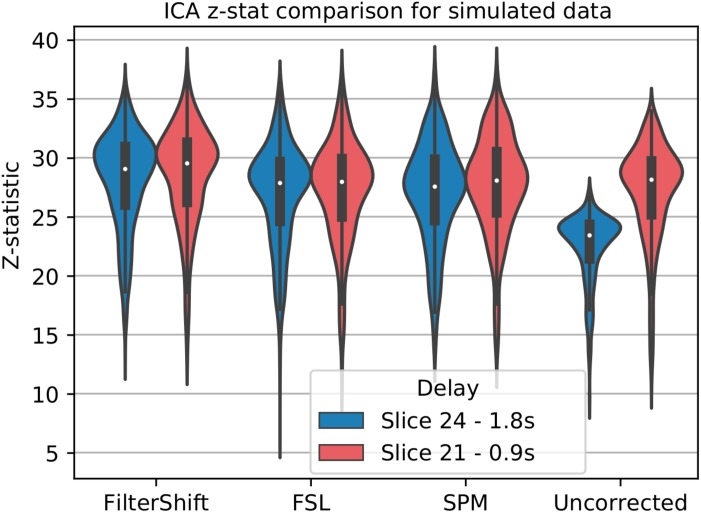
STC on the ability to extract functionally connected regions in simulated data. For each simulated subject, a task IC was extracted with MELODIC, and the IC’s time series was regressed onto the subject’s data using FSL’s dual regression. Z statistics from a low and high delay slice were extracted for every subject and plotted here. For uncorrected data, the extracted IC performs as well as STC data in the low delay slice, but poorly explains the variance in the high delay slice, resulting in a lower z-statistic. For STC data, the average z-statistics are comparable for both high and low delay slices.

**FIGURE 12:**
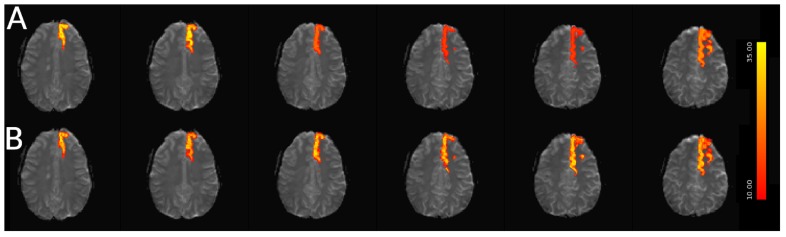
Spatial map of functionally connected regions with and without STC on simulated data. The left Superior Frontal region is extracted using ICA on both STC data and uncorrected data. The ICA from each dataset is then regressed back on the original data for **(A)** uncorrected and **(B)** FSL STC data. In the left-most slices, regression z-statistics are higher in the uncorrected data, however, overall the STC data best describes the variance in the entire region, as seen by the drop in z-statistics in slices 3–5 in uncorrected data, while the STC data’s z-statistics remain high across all slices.

For simulated data, we extract and plot significant z statistics from a slice with higher temporal delay, and a slice with low temporal delay (slice 20 and 23) for all simulated subjects in Figure 11. For the low delay slice, we see that all STC methods perform similarly, and the extracted z-statistics are relatively the same. For the high delay slice, we see that the uncorrected data has lower z-stats than the low delay slice. However, STC improves the statistics for the high delay slice in all cases. This can be seen in Figure 12, where ICA dual regression is run on both uncorrected data, shown in A, and STC data shown in B, with the resulting z-statistics shown for six consecutive slices. The IC for the LSF region covers the same spatial region in both analysis, however, after performing dual regression, we see that for uncorrected data, the time series of the IC only strongly predicts the fMRI signal in some of the slices, while for the STC data, the z-statisitcs remain high across all slices. Across all subjects and slices, STC was able to improve the z-statistics of the high delay slice.

**FIGURE 13:**
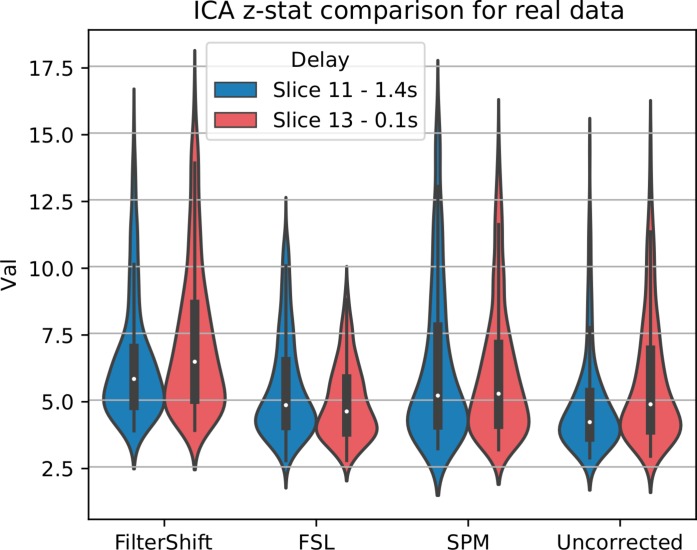
STC on the ability to extract functionally connected regions in real data. For each real subject, a task IC was extracted with MELODIC, and the IC’s time series was regressed onto the subject’s data using FSL’s dual regression. Z statistics from a low and high delay slice were extracted for low motion subjects and plotted here. For uncorrected data, the extracted IC performs as well as STC data in the low delay slice, but poorly explains the variance in the high delay slice, resulting in a lower z-statistic. For STC data, the average z-statistics are comparable for both high and low delay slices.

For real data, we repeated the analysis done for simulated data, and extracted z-statistics for two slices with low and high delay from all subjects (Slice 15 and 18), and plotted the distributions in Figure 13. As seen in this figure, the differences between slices have largely vanished, across all subjects and slices, z-statisitcs increased greatly with FS by 27% compared to FSL, 18% compared to SPM, and 30% compared to uncorrected data.

## Discussion

Our previous work has demonstrated the benefit of STC on fMRI data in both real and simulated data which had already been shown in the literature (Henson et al., 1999; Calhoun et al., 2000; Vogt et al., 2009; Sladky et al., 2011). However, because the slice-timing problem is fundamentally intertwined with many confounding factors such as involuntary head motion during scanning, spatial realignment, 3D smoothing, and spatial normalization, it has as yet been difficult to determine whether STC would provide any benefit to the analysis. In the current study, we rigorously examined the impact of these major confounding factors on the effectiveness of STC. We showed that even though the effectiveness of STC changes depending on the preprocessing pipeline, it still remains beneficial to be included as a preprocessing step. While focusing on 20 highly significant voxels does limit our conclusions about STC to only true positives, previous studies have shown that STC does not adversely affect parameter estimate bias, and in fact was shown to suppress bias, so we do not anticipate the need to evaluate STC on voxels outside our ROIs (Sladky et al., 2011). Although we cannot determine from this study if the conclusions reached here generalize over the whole brain, we have isolated the voxels most affected by STC, and so if an effect is not found here, it is unlikely to be found anywhere else. Other studies on STC have shown the benefit of STC in regions distributed across the brain (Henson et al., 1999; Calhoun et al., 2000; Sladky et al., 2011), and so it is likely the observations made here will apply to at least the entire region of activation, if not the whole brain, though the magnitude of the effect will change with the temporal delay of the slice.

There are additional STC methods not examined in this paper. Some studies attempt to combine both MC and STC using 4D interpolation to account for temporal and spatial misalignment in one step (Roche, 2011). This approach takes into account both the position of the head in space as well as the timing of each slice. Other methods have proposed slice-by-slice realignment and resampling to a standard template (Bannister et al., 2007). This method tracks which slices contribute to each voxel, and interpolation is carried out using the known slice acquisition times. While these methods seem to be addressing the issue of interaction between STC and MC, it is crucial to emphasize that the EPI sequence in which fMRI data are acquired is a steady-state acquisition and any disruption in its equilibrium (i.e., over/under excitation due to motion) will be broadcasted to other slices and volumes and it will take multiple acquisitions until it reaches to its steady state again (Power et al., 2015). Any fMRI volume acquired during this period will be contaminated which essentially deteriorates the effectiveness of any simultaneous STC and MC.

### Pipeline Order Without Spatial Smoothing

We analyzed the interaction of STC, MC, and their order in the pre-processing pipeline on different levels of motion. In simulated data, the significance of pipeline order seemed to be more effected by the interleave parameter rather than motion level. Real data seemed to indicate that there was no difference in the order for low and high motion cases, however, it’s beneficial to apply motion correction first for the medium motion case for FSL and SPM. Previous work has also found differences in pipeline performance for subjects with different levels of motion (Churchill et al., 2012). As our real data has an interleave parameter of 6, these findings are not replicated in the simulated data, which showed no significant differences between pipeline order. The discrepancies between the real and simulated results may be from the fact that all simulated data used the same subject morphology. Because of this, there is no inter-subject variability across the simulated brains. This essentially makes the comparison one-to one, whereas for real data it is impossible to get to such correspondence in the selected voxels for comparison. Another factor that could contribute to these differences is an incomplete simulation of motion on our part, as motion has been known to create non-linear artifacts in the scanner that are difficult to characterize, but become non-linearly larger with greater head motion (Power et al., 2012). We also don’t include spin history effects or B0 inhomogeneity in our simulation, both of which have motion interactions (Drobnjak et al., 2006). Our simulation captured rigid body motion as well as temporal shifts due to slice acquisition. The fact that our simulated results are so different from our real results simply means that there are more artifacts caused by motion than just moving-head artifacts, simulated here, and the interaction of motion and slice timing may be more complicated than simply linearly adding the effects of both. While this is in some ways a limitation, it also gives us insight to the effectiveness of some pre-processing methods. For example, because motion parameter regression works so well for our simulated data, it is possible that this technique is good at removing variance caused by partial volume effects induced by motion. More research should be done in future to investigate this possibility.

In real data, motion artifacts work in two ways to contaminated fMRI signal: it introduces fluctuations in the signal that would both increase the variance, as well as decrease the *z*-score of the time series from the task-timing, weakening the beta estimate. This may also explain why it is only better to apply motion correction first in medium motion cases for our real data. For low motion, there is very little correction to be made, and so the actual application of MC has only a small effect on the data, and therefore has an insignificant effect on the z-scores. For high motion, the non-linear artifacts from motion may be large enough that traditional rigid body realignment is unable to correct for them. This would mean that regardless of when MC or STC is applied, there are large errors in the data that remain uncorrected, keeping the *z*-scores low. For medium motion, the motion may be large enough to benefit from rigid body realignment, while small enough to not induce significant non-linear artifacts, which would result in the maximum benefit of its application. Regardless of the amount of motion, our results suggest that it is always beneficial to apply both STC and spatial realignment. The benefit of applying them both, in any order, is much greater than the benefit of applying only one. Even for low motion subjects, the average *z*-scores from data with both MC and STC were greater than those from just MC alone.

In general, these results highlight how dependent STC and MC are on each other. In Simulated data, only performing STC was better than only performing MC for low motion subjects, however, in high-motion simulated data we find just the opposite is true, hinting that motion destroys the ability of STC to temporally realign the BOLD signals. One possibility could be that brain regions may shift from one slice to another due to motion. Because each slice is sampled at a different offset, even after spatial realignment, the time series will be made up of temporally non-uniform samples, which current STC routines cannot correct for.

While this doesn’t untangle the complicated interactions between STC and motion, it does provide a quantitative measure to aid in pre-processing techniques. Recently, new preprocessing techniques have attempted to reconcile these interactions (Jones et al., 2008; Beall and Lowe, 2014). Jones et al. (2008) uses a method that involves quantifying which native space slice each voxel came from in the registered standard space volume. By taking into account the true sampling delay of each voxel, an accurate voxel-wise estimate of physiological noise can be created to account for such delays. By using a similar technique to identify the true time of acquisition of any given voxel in a registered volume, it may be possible to reduce the slice timing/motion interaction to a non-uniform sampling and reconstruction problem. Future work could involve examining the efficacy of such a model.

The dice overlap for simulated data shown in Figure 6 indicates that as motion increases, the order in which STC and MC are applied becomes more important with regard to the voxel overlap. This can be seen by comparing the “Before MC” to the “After MC” columns. The more similar these columns are, the less important pipeline order is for spatial overlap. MC before STC has a higher dice overlap than when STC is applied first. One possible reason for this is because the SR has no STC, and the shifts are accounted for in the regression itself. This could be seen as “MC before STC,” since the timing is accounted for after MC. Assuming that the SR method does successfully identify the voxels with the highest signal, we can infer that higher overlap with this method is related to a better pipeline. Interestingly, uncorrected data seems to be able to maintain the location of the top 20 voxels even in high motion, however from Table 1, we know that the *z*-score of these voxels are significantly reduced. For real data shown in Figure 7, we see that our method becomes more reliant on MC for a high spatial overlap as the motion level increases (“No MC” columns compared to any other column). In real data, we see that the effect of the order of STC and MC is smaller. We also see a significant drop in dice overlap when MC is not applied. This further highlights the importance of performing both STC and MC. Further, STC seems to benefit voxel overlap more than MC. The effect of No MC can be seen by comparing the “Uncorrected” bar to any other bar in the “No MC” column. Only MC is applied to “uncorrected data” in the “Before MC” case, so the effect of MC can be seen by comparing the “Uncorrected” bar in the “No MC” column with the “uncorrected” bar in the “Before MC” column. “No MC” means no MC has been applied, and so for uncorrected data, neither STC nor MC is performed in this category.

Prewhitening had no significant effect on STC in real data. In addition, our results are in line with the existing literature. It has been demonstrated in Olszowy et al. (2019) that STC has only a small impact on autocorrelation, and did not significantly influence the results when combined with PW. Combining PW and MC did effect the results, and this result was modulated by motion. It has been shown previously that motion correction can induce temporal correlations between voxels (Power et al., 2012). It’s possible that these motion artifacts are also inducing autocorrelation within a voxel. Understanding this phenomenon would be valuable to the field, and is left as an area of future research.

The reliability measures should not be taken as evidence that there are no differences between pipelines. The BOLD signal only makes up about 5% of the variance in fMRI. For a given task, the vast majority of the voxels are only noise, and have no relevant signal in their time series. Applying a time shift on these noisy voxels will have no effect on the reliability. Of the voxels that are activated, only those that fall on high delay slices will see significant changes due to STC. Because of this, the reliability measure will be made up of mostly voxels that have little to no change due to slice timing correction, with only a small portion seeing any real difference. This is why we wouldn’t expect to see a difference in the reliability measure, and it is for this reason that we focused our analysis on voxels with high *t*-values in high-delay slices for all investigations in the paper.

### Motion Parameter Residualization

Motion parameter residualization lowered the *z*-score slightly in most cases for both real and simulated data, though the effects were larger for simulated data. This indicates that the presence of motion parameter estimates in the GLM likely interfere with accurate beta estimation. This can be caused by colinearity between the motion parameters and the regressor being used in the analysis. When using MPR, in simulated data, STC before MC was extremely detrimental to the beta value estimation. This could be because STC effects the accurate estimation of motion parameters (Power et al., 2017). It is possible that the results for STC before MC would improve if the parameters are estimated first, and MC itself is performed after STC. There are many cases where “No MC” performs almost as well as other pipelines, again highlighting the fact that our simulated data does not add non-linear motion artifacts to the data, therefore linear regression of motion parameters should be sufficient for removing most of the variance. It’s also important to note that our analysis was performed on highly significant voxels, which tend to fall inside the ROI, rather than along the edges. It’s likely that motion has a larger effect on voxels along the edges of activation, as they will be more susceptible to sampling a new brain region with no task-related signal when the subject moves.

For real data, the low motion subjects saw a decrease in statistics with the addition of motion parameter regression, indicating that the added regressors either failed to model the low motion artifacts, or there simply wasn’t enough motion-induced variance to make up for the loss in degrees of freedom. If the motion in the scan is simply too low for an accurate estimate, the realignment parameters would be essentially noise. Real data with MPR and no STC performed significantly worse than all methods with STC for medium and high motion. It’s possible that the motion parameters themselves are sensitive to time shifts.

Our results agree with Churchill et al., who found that MPR was generally unhelpful, and in fact harmful for low motion subjects, in the sense that it lowered their chosen measurement of quality in that paper (Churchill et al., 2012). However, it is difficult to compare our motion levels with this study, as FWD is not used in their study, and “high motion” is based only on the extracted yaw parameters.

### Spatial Smoothing on STC

For spatial smoothing, the achieved STC gain became very small with a large kernel size (>6.5 mm). For real data, differences between STC methods disappeared with a 5 mm smoothing kernel, and at 8 mm STC data is indistinguishable from uncorrected data for low and medium motion subjects. It has been shown that the optimal FWHM varies between 6 and 12 mm, depending on the experimental tasks and the statistics of interest (Mikl et al., 2008). It is important to note that at this level removing the STC step might slightly improve or worsen the results, depending on the location of a given ROI, as shown by the *z*-score of the “uncorrected” column of the low motion subjects. This is rather counter-intuitive and might be considered as evidence that STC is not beneficial at all. However, recent studies such as the HCP has also taken a critical look at smoothing and its effects on neuroimaging data, and has greatly restricted the size of the kernel, as well as the extent of the areas used in smoothing (Glasser et al., 2013), which may be enough to retain the benefits of STC.

As in the real data, mean *z*-score from simulated uncorrected data only decreased monotonically for sequential acquisition and interleave 2 acquisitions. For interleave 6 acquisition, the initial improvement seen in the 3.5, 5, and 6.5 mm kernels are caused by the phenomenon described in the “Introduction,” where time series from adjacent slices with less delay actually improve the statistics of the high delay slice. Initially, this benefits the *z*-score, shown in the 3.5 mm kernel case, as it averages the data with voxels in adjacent slices. These voxels are still in the LSF region, but with lower temporal delay. This initial increase is reduced as the kernel size continues to grow, because the LSF region is just a narrow (less than 5 mm) cortical region, which cannot extend beyond two voxels in the x/y plane. Increasing the kernel size beyond 3.5 mm will cause the blending of the voxels outside the LSF that have completely different underlying BOLD signal, which essentially corrupts the time series, reducing the *z*-score. These results do not suggest that spatial smoothing should not be performed, but rather they show how smoothing interacts with, and may obscure, the effects of precise preprocessing steps such as STC.

Finally, it is important to note the failure of slice-based regressors (SR) method, as the gold standard, in the presence of smoothed data. This method performs worse than FS in simulated data with interleave 2 and interleave 6 with the 8 mm kernel, and for real data it performs worse than all other STC methods for all motion levels with the 8mm kernel. This is due to the fact that in our gold standard, the regressors are accounting for a slice dependent delay. Spatial smoothing averages a slice with time series from the surrounding slices that fall within the kernel. Each adjacent slice was sampled with a different temporal delay. Smoothing this time series with data from surrounding slices acquired at different offsets greatly contaminates the fMRI signal, and eliminates any benefit gained by using slice-specific regressors. Additionally, in our gold standard pipeline, the data was filtered with the same low-pass filter as used in the FS method without performing any temporal shifting on the data. If this filtering was not performed, the SR method would likely be even worse than FSL and SPM’s STC techniques (which only apply very slight low-pass filtering, inherent to their interpolation kernels). This suggests that slice-based regressions should not be used with data that has undergone spatial smoothing.

### STC on Short TRs

Both our simulated and real data are based on event-related design, which requires fast (<5 s) and random stimuli duration. Block-designed experiments, on the other hand, have much longer (>10 s) stimulus duration, thus making it less susceptible to the acquisition offset delay. It has already been shown in the literature that block design accompanied with moderate 3D smoothing may not benefit substantially from STC (Sladky et al., 2011). Therefore, we decided not to repeat that experiment again in this work. Nevertheless, extremely high TRs (TR > 5 s) without STC could not be tolerated even for block related designs. On the other hand, it is generally assumed that sub-second TR data will not benefit from STC. We have shown with our results that FS STC does improve *z*-score, even on multi-band data. The increased number of time points only makes the regressors more sensitive to temporal shifts and interpolation errors. Because our method performs a more accurate reconstruction than traditional STC techniques, we are able to significantly improve the *z*-score in these high frequency data sets. Furthermore, a short TR may reduce aliasing of physiological noise into the frequency range of the BOLD signal. Our STC method can potentially filter out more of the high frequency noise, and retain only the range of frequencies in which the BOLD signal is biologically plausible. This significantly improves *z*-score beyond what traditional STC is capable of.

### Effect of STC on Functional Connectivity Analysis

We have shown that FC network extraction can benefit from STC. In simulated data, we clearly show the problem faced when extracting task based networks from data without STC. The difference in z-statistics between slices with large temporal offsets is observable in the uncorrected data, but is completely corrected for in STC data. This is shown quantitatively in Figure 11 and qualitatively in Figure 12. From these figures, we can see that the extracted IC’s time series “locked” strongly with slices of a certain temporal delay. Even a voxel in the same region with the same time course, but sampled at an offset, will be assigned a weaker weight to that IC. That IC’s time series will also account for less variance in the dual regression, resulting in lower z-statistics. The reason we don’t see the difference between high and low delay slices in real uncorrected data is partly due to inter-subject variability in region activation. Additionally, there’s no guarantee that MELODIC will always extract an IC with a time series aligned to the low-delay slices. If the variability between subjects is enough to randomly distribute the slices with which the IC correlates more strongly to (High delay slices or low delay slices), then we would expect the group level difference to become smaller and smaller as we add subjects to the analysis. For example, one subject’s IC might be more correlated with a high delay slice, while the other subject’s IC might be more correlated with a low delay slice. These differences would average out, and we wouldn’t see the dramatic delay effect that we do in simulated data. It’s true that there is also no guarantee that MELODIC would consistently prefer low-delay slices for simulated data, however, given the identical morphology, activation region, and low motion, we effectively minimized all non-signal related sources of variance, so we would expect similar results.

Despite these potential confounds, we are still able to show that STC, specifically FS, increases the statistical fit of the IC’s extracted time series to the data in both real and simulated subjects. By removing sources of variance such as temporal offsets, voxels are more strongly correlated with each other, resulting in a better network extraction, as well as a more accurate estimate of the network’s time series.

## Conclusion

In an effort to critically examine the effects of STC in the context of a standard preprocessing pipeline, we have performed a series of experiments in which we manipulate the preprocessing pipeline on data with various levels of motion and with various slice acquisition orders. While no clear rule for the order of pre-processing steps emerged, we were able to reinforce the importance of both STC and MC. Here, we summarize the key findings from our experiments: For all levels of motion and for all slice acquisition orders, STC and MC together significantly improved z-scores in both real and simulated data. MPR altered nearly all simulated cases, as well as all medium and high motion cases for real subjects. For real data, MC before STC was always the optimal pipeline when using MPR, likely due to the fact that a more accurate motion estimate can be obtained if MC is performed first (Power et al., 2017). However, this nuisance regression proved detrimental for all *z*-scores across all scan conditions. We found that on multiband data with extremely short TRs, STC significantly improved results. Importantly, we found that STC of any kind never lowered the resulting *z*-scores. The only case that showed minimal improvement from STC was data filtered with large smoothing kernels. However, in studies using smaller smoothing kernels, such as in surface based analysis, STC will only become more beneficial as the smoothing kernels shrink. Finally, we found that STC improves the coherence across slices of FC networks extracted from task-based fMRI data using ICA. STC improved the fit of the IC to the time series’ of voxels in the IC, especially those in high delay slices.

This paper provided an in-depth look at many common situations in which STC may be implemented. We have shown that in all such cases, STC is a valuable addition to the preprocessing pipeline.

## Data Availability

The datasets generated for this study are available on request to the corresponding author.

## Author Contributions

DP ran all the analysis, generated the figures, and wrote the manuscript. QR generated the simulated data set and wrote the manuscript.

## Conflict of Interest Statement

The authors declare that the research was conducted in the absence of any commercial or financial relationships that could be construed as a potential conflict of interest.
