# Supplementary figures and images for: The Benefit of Slice Timing Correction in Common fMRI Preprocessing Pipelines
Source: Front Neurosci. 2019 Aug 20;13:821. doi: 10.3389/fnins.2019.00821 (PMC6736626; doi:10.3389/fnins.2019.00821)

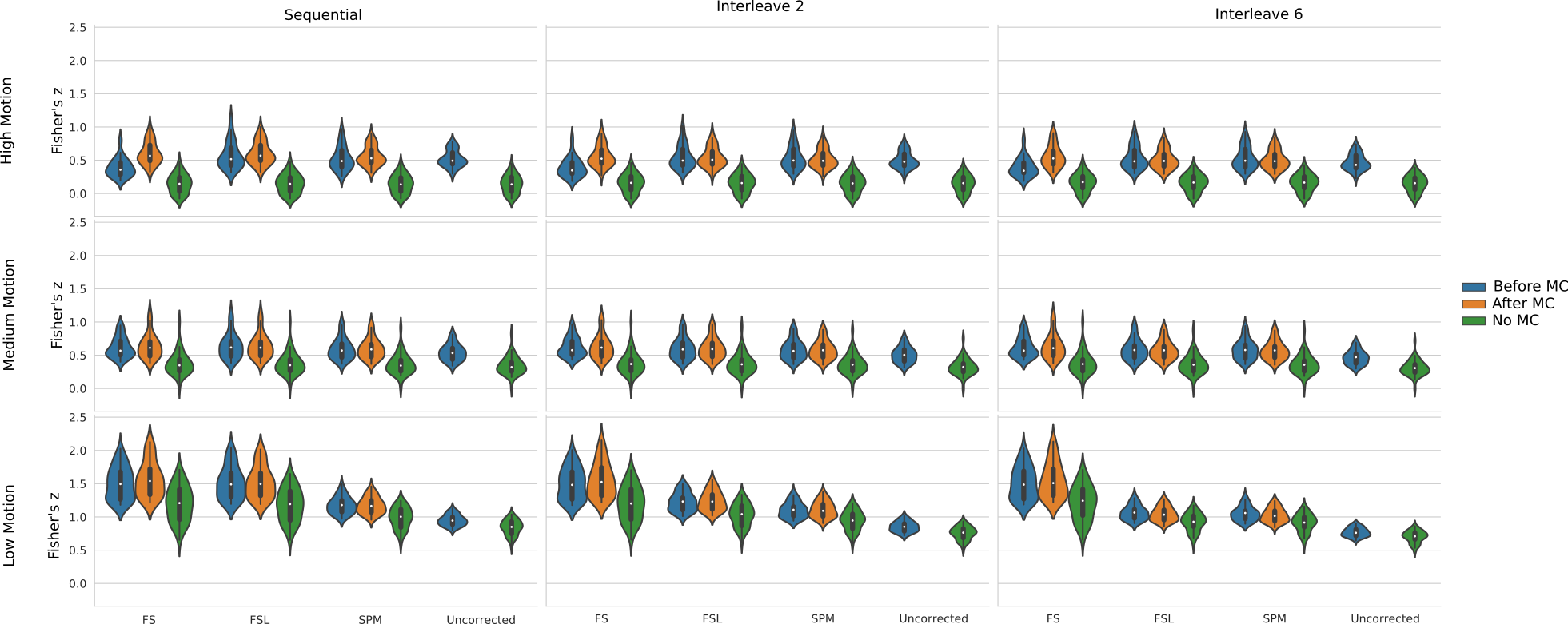

Supplement: Supplementary file 1 [file Image_1.TIF]

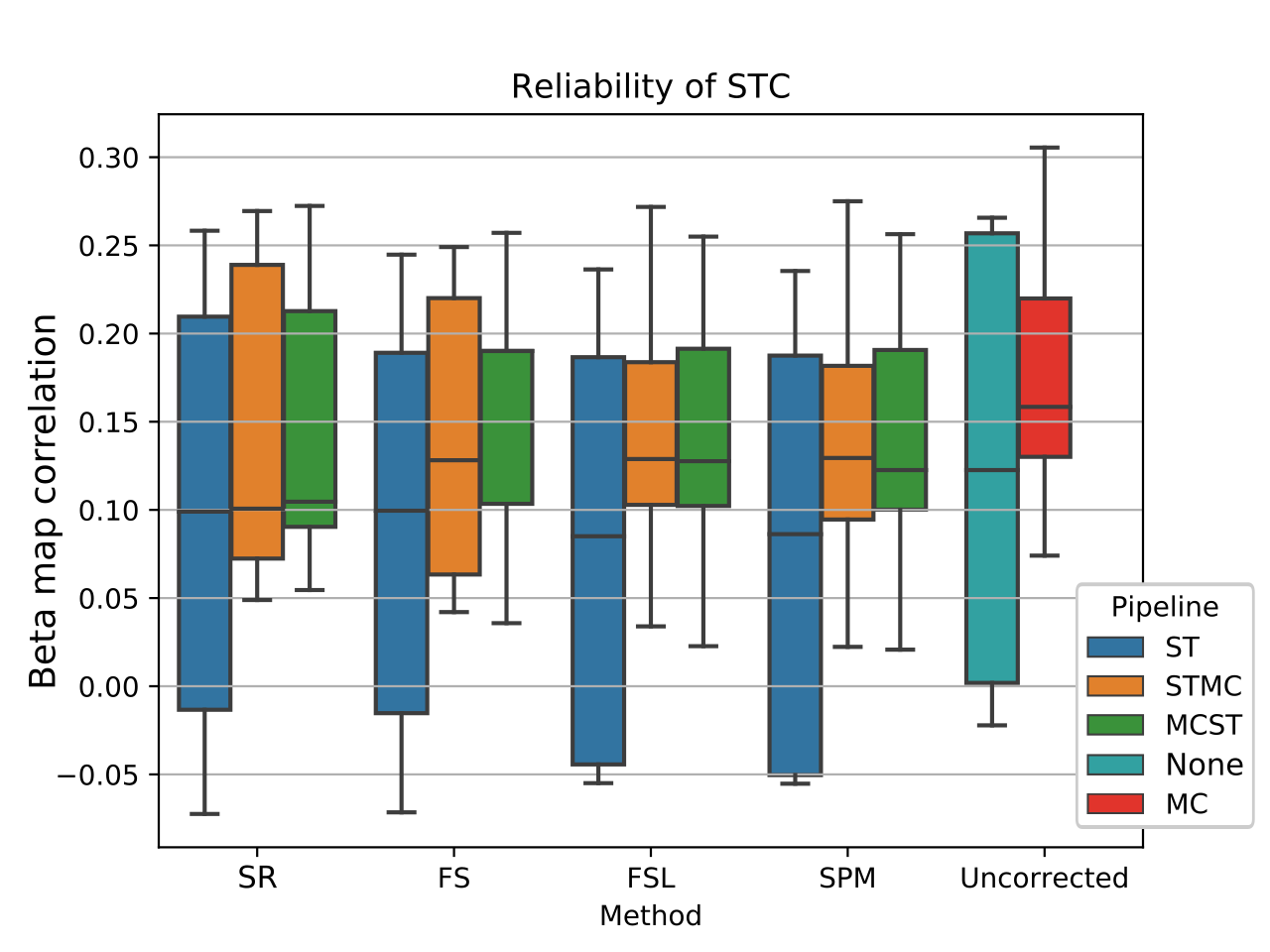

Supplement: Supplementary file 2 [file Image_2.TIF]

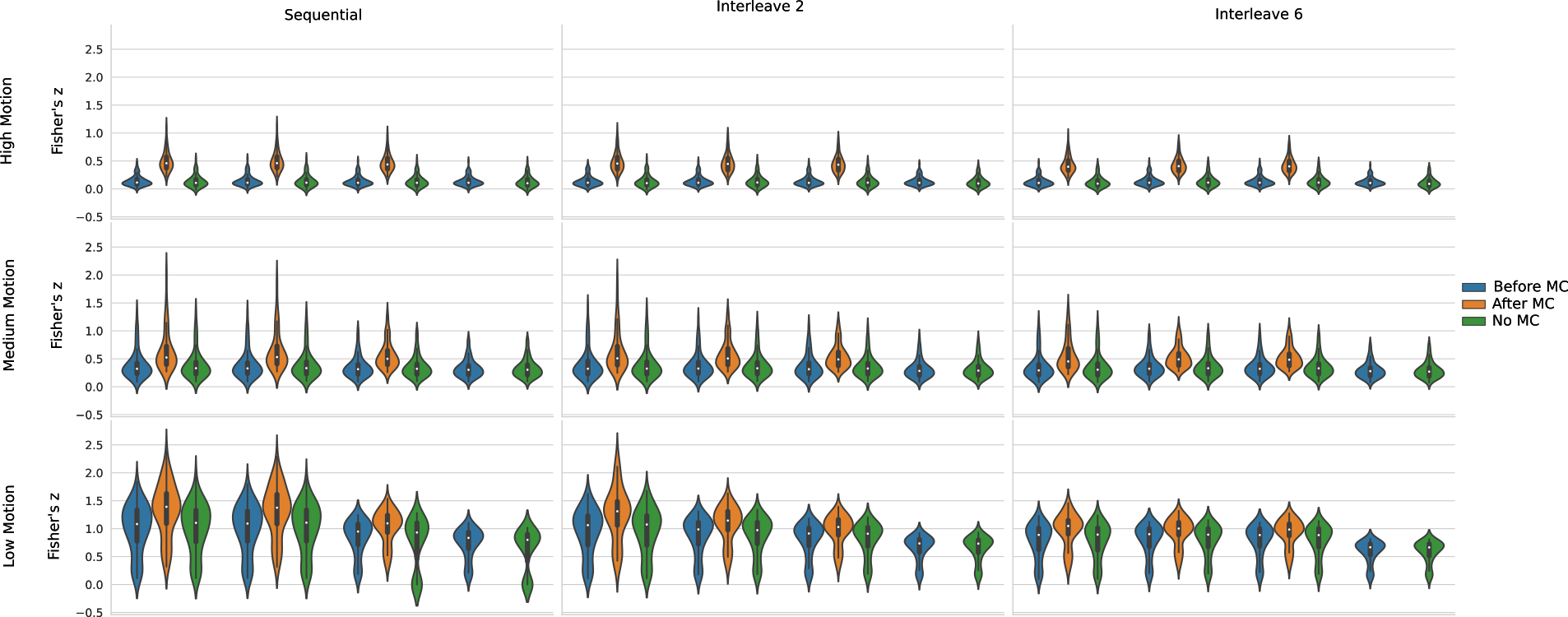

Supplement: Supplementary file 3 [file Image_3.TIF]

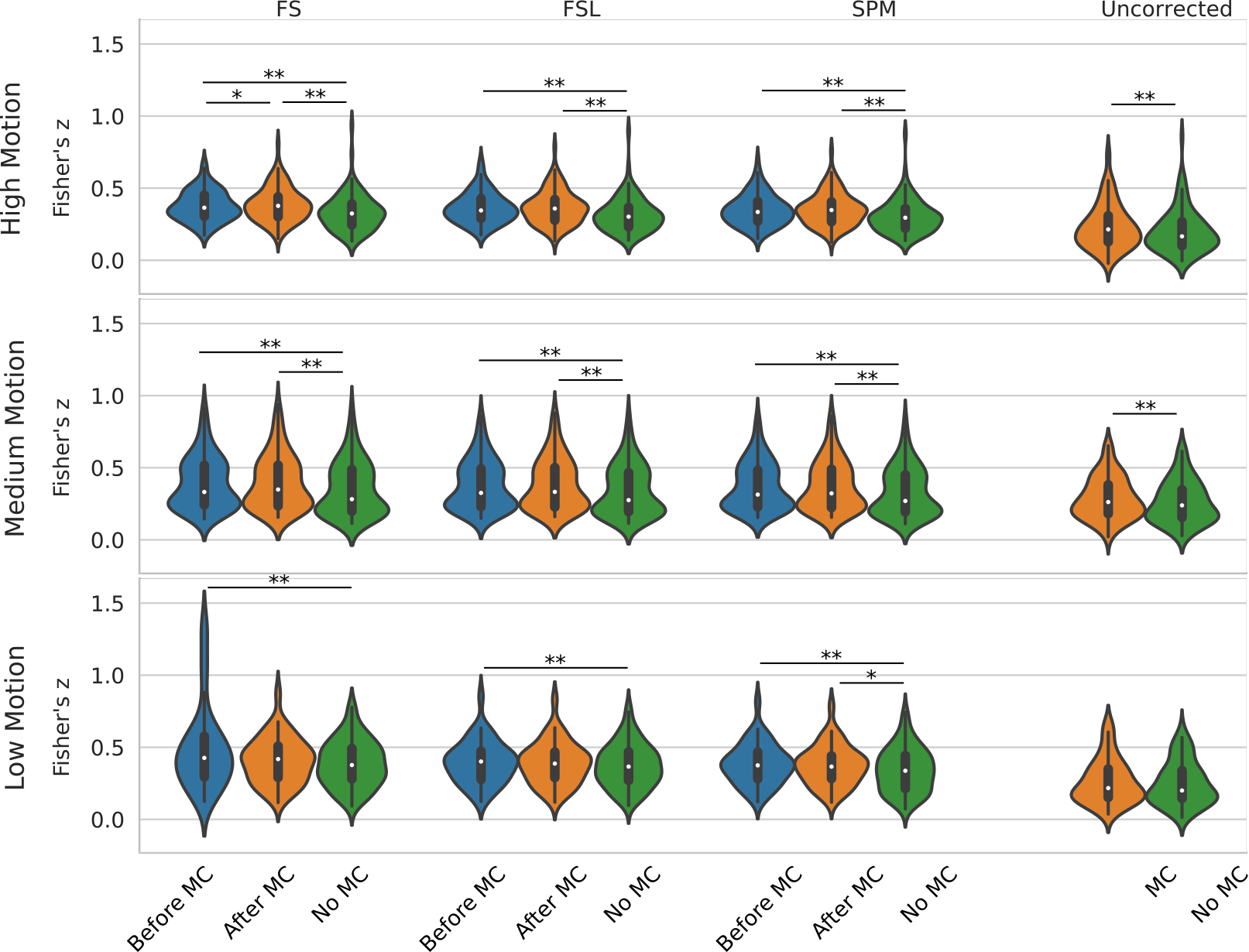

Supplement: Supplementary file 4 [file Image_4.TIF]
