# Supplementary material for: The Benefit of Slice Timing Correction in Common fMRI Preprocessing Pipelines
Source: Front Neurosci. 2019 Aug 20;13:821. doi: 10.3389/fnins.2019.00821 (PMC6736626; doi:10.3389/fnins.2019.00821)
Supplement: Supplementary file 5 [file Table_1.DOCX]

Figure S1. **Effect of the order in which STC and MC are applied in the preprocessing pipeline in simulated data.** Mean z-score of top 20 voxels identified in the Shifted Regressor “Before MC” case, extracted across various preprocessing pipelines for 5 simulated subjects, as shown in table 1. Significant differences are indicated in the corresponding table.

Figure S2. **The effect of STC on fMRI reliability.** The reliability of our fMRI analysis is calculated for each pipeline and STC method. Reliability is defined as the correlation of the beta maps that are generated when the timeseries is split in half, and the GLM is run on each segment. No significant differences were found whether STC was performed or not. Additionally, there were no significant differences between pipelines within STC methods.

Figure S3. **Effect of the order in which STC and MC are applied in the preprocessing pipeline in simulated data with MPR.** Mean z-score of top 20 voxels identified in the Shifted Regressor “Before MC” case, extracted across various preprocessing pipelines for 5 simulated subjects with motion parameter risidualization, as shown in table 2. Significant differences are indicated in the corresponding table.

Figure S4. **Effect of the order in which STC and MC are applied in the preprocessing pipeline in real data with MPR.** Z-scores from the top 20 voxels from a visual ROI of 30 real subjects’ time series using different preprocessing pipelines, with no smoothing. FS, FSL, and SPM slice timing correction were applied before motion correction, after motion correction, or without any motion correction. For the “Uncorrected” case, only motion correction was applied in the preprocessing pipeline.
